# Evolutionary heritage influences Amazon tree ecology

**DOI:** 10.1098/rspb.2016.1587

**Published:** 2016-12-14

**Authors:** Fernanda Coelho de Souza, Kyle G. Dexter, Oliver L. Phillips, Roel J. W. Brienen, Jerome Chave, David R. Galbraith, Gabriela Lopez Gonzalez, Abel Monteagudo Mendoza, R. Toby Pennington, Lourens Poorter, Miguel Alexiades, Esteban Álvarez-Dávila, Ana Andrade, Luis E. O. C. Aragão, Alejandro Araujo-Murakami, Eric J. M. M. Arets, Gerardo A. Aymard C, Christopher Baraloto, Jorcely G. Barroso, Damien Bonal, Rene G. A. Boot, José L. C. Camargo, James A. Comiskey, Fernando Cornejo Valverde, Plínio B. de Camargo, Anthony Di Fiore, Fernando Elias, Terry L. Erwin, Ted R. Feldpausch, Leandro Ferreira, Nikolaos M. Fyllas, Emanuel Gloor, Bruno Herault, Rafael Herrera, Niro Higuchi, Eurídice N. Honorio Coronado, Timothy J. Killeen, William F. Laurance, Susan Laurance, Jon Lloyd, Thomas E. Lovejoy, Yadvinder Malhi, Leandro Maracahipes, Beatriz S. Marimon, Ben H. Marimon-Junior, Casimiro Mendoza, Paulo Morandi, David A. Neill, Percy Núñez Vargas, Edmar A. Oliveira, Eddie Lenza, Walter A. Palacios, Maria C. Peñuela-Mora, John J. Pipoly, Nigel C. A. Pitman, Adriana Prieto, Carlos A. Quesada, Hirma Ramirez-Angulo, Agustin Rudas, Kalle Ruokolainen, Rafael P. Salomão, Marcos Silveira, Juliana Stropp, Hans ter Steege, Raquel Thomas-Caesar, Peter van der Hout, Geertje M. F. van der Heijden, Peter J. van der Meer, Rodolfo V. Vasquez, Simone A. Vieira, Emilio Vilanova, Vincent A. Vos, Ophelia Wang, Kenneth R. Young, Roderick J. Zagt, Timothy R. Baker

**Affiliations:** 1 School of Geography, University of Leeds, Leeds LS2 9JT, UK; 2 School of Geosciences, University of Edinburgh, 201 Crew Building, King's Buildings, Edinburgh EH9 3FF, UK; 3 Royal Botanic Garden Edinburgh, 20a Inverleith Row, Edinburgh EH3 5LR, UK; 4 Université Paul Sabatier CNRS, UMR 5174 Evolution et Diversité Biologique, bâtiment 4R1, Toulouse 31062, France; 5 Jardín Botánico de Missouri, Prolongacion Bolognesi Mz. E, Lote 6, Oxapampa, Pasco, Peru; 6 Forest Ecology and Forest Management Group, Wageningen University and Research, PO Box 47, 6700 AA Wageningen, The Netherlands; 7 School of Anthropology and Conservation, University of Kent, Marlowe Building, Canterbury, Kent CT2 7NR, UK; 8 Fundación Con Vida, Medellin Cra 48: 20-114, Colombia; 9 Biological Dynamics of Forest Fragment Project (INPA & STRI), C.P. 478, Manaus, Amazonas 69.011-970, Brazil; 10 Geography, College of Life and Environmental Sciences, University of Exeter, Drive, Exeter, Rennes EX4 4RJ, UK; 11 National Institute for Space Research (INPE), São José dos Campos, São Paulo, Brazil; 12 Museo de Historia Natural Noel Kempff Mercado, Universidad Autonoma Gabriel Rene Moreno, Casilla 2489, Av. Irala 565, Santa Cruz, Bolivia; 13 Alterra, Wageningen University and Research Centre, PO Box 47, Wageningen 6700 AA, The Netherlands; 14 UNELLEZ-Guanare, Programa del Agro y del Mar, Herbario Universitario (PORT), Mesa de Cavacas, Estado Portuguesa 3350, Venezuela; 15 International Center for Tropical Botany, Department of Biological Sciences, Florida International University, Miami, FL 33199, USA; 16 Universidade Federal do Acre, Campus de Cruzeiro do Sul, Acre, Brazil; 17 INRA, UMR 1137 ‘Ecologie et Ecophysiologie Forestiere’, Champenoux 54280, France; 18 Tropenbos International, PO Box 232, Wageningen 6700 AE, The Netherlands; 19 National Park Service, 120 Chatham Lane, Fredericksburg, VA 22405, USA; 20 Smithsonian Institution, 1100 Jefferson Dr, SW, Washington, DC 20560, USA; 21 Proyecto Castaña, Madre de Dios, Peru; 22 Centro de Energia Nuclear na Agricultura, Universidade de São Paulo, São Paulo, Sao Paulo, Brazil; 23 Department of Anthropology, University of Texas at Austin, SAC Room 5.150, 2201 Speedway Stop C3200, Austin, TX 78712, USA; 24 Universidade do Estado de Mato Grosso, Campus de Nova Xavantina, Caixa Postal 08, 78.690-000, Nova Xavantina, Mato Grosso, Brazil; 25 Department of Entomology, Smithsonian Institution, PO Box 37012, MRC 187, Washington, DC 20013-7012, USA; 26 Museu Paraense Emilio Goeldi, C.P. 399, 66.040-170, Belém, Pará, Brazil; 27 Cirad, UMR EcoFoG (AgroParisTech, CNRS, Inra, U Antilles, U Guyane), Campus Agronomique, Kourou 97310, French Guiana; 28 Centro de Ecología IVIC, Caracas, Venezuela; 29 Institut für Geographie und Regionalforschung, University of Vienna, Wien, Austria; 30 INPA, Av. André Araújo, 2.936 - Petrópolis – 69.067-375, Manaus, Amazonas, Brazil; 31 Instituto de Investigaciones de la Amazonia Peruana, Apartado 784, Iquitos, Peru; 32 AGTECA - Amazónica, Santa Cruz, Bolivia; 33 Centre for Tropical Environmental and Sustainability Science (TESS) and College of Science and Engineering, James Cook University, Cairns, Queensland 4878, Australia; 34 Department of Life Sciences, Imperial College London, Silwood Park Campus, Buckhurst, Road, Ascot, Berkshire SL5 7PY, UK; 35 Environmental Science and Policy, and the Department of Public and International Affairs, George Mason University (GMU), Washington, DC, USA; 36 Environmental Change Institute, School of Geography and the Environment, University of Oxford, Oxford, UK; 37 Programa de Pós-graduação em Ecologia e Evolução, Universidade Federal de Goias, Goiânia, Goias, Brazil; 38 Escuela de Ciencias Forestales, Unidad Académica del Trópico, Universidad Mayor de San Simón, Sacta, Bolivia; 39 Universidad Estatal Amazónica, Puyo, Pastaza, Ecuador; 40 Universidad Nacional de San Antonio Abad del Cusco, Av. de la Cultura N° 733, Cusco, Peru; 41 Universidad Técnica del Norte and Herbario Nacional del Ecuador, Casilla 17-21-1787, Av. Río Coca E6-115, Quito, Ecuador; 42 Universidad Regional Amazónica IKIAM, Tena, Ecuador; 43 Broward County Parks and Recreation Division, 950 NW 38th St., Oakland Park, FL 33309, USA; 44 Center for Tropical Conservation, Duke University, PO Box 90381, Durham, NC 27708, USA; 45 Doctorado Instituto de Ciencias Naturales, Universidad ciol de Colombia, Colombia; 46 Instituto de Investigaciones para el Desarrollo Forestal (INDEFOR), Facultad de Ciencias Forestales y Ambientales, Universidad de Los Andes, Conjunto Forestal, C.P. 5101, Mérida, Venezuela; 47 Department of Geography and Geology, University of Turku, 20014 Turku, Finland; 48 Museu Universitário, Universidade Federal do Acre, Rio Branco, AC 69910-900, Brazil; 49 Institute of Biological and Health Sciences (ICBS), Federal University of Alagoas, Maceió, AL, Brazil; 50 Naturalis Biodiversity Center, Vondellaan 55, Postbus 9517, Leiden 2300 RA, The Netherlands; 51 Iwokrama Intertiol Centre for Rainforest Conservation and Development, 77 High Street Kingston, Georgetown, Guyana; 52 Van der Hout Forestry Consulting, Jan Trooststraat 6, Rotterdam 3078 HP, The Netherlands; 53 School of Geography, University of Nottingham, University Park, Nottingham NG7 2RD, UK; 54 Van Hall Larenstein University of Applied Sciences, PO Box 9001, 6880 GB Velp, The Netherlands; 55 Universidade Estadual de Campinas, Núcleo de Estudos e Pesquisas Ambientais – NEPAM, Campinas, São Paulo, Brazil; 56 Facultad de Ciencias Forestales y Ambientales, Universidad de Los Andes, Mérida, Venezuela; 57 Centro de Investigación y Promoción del Campesinado - regional Norte Amazónico, C/ Nicanor Gonzalo Salvatierra N° 362, Casilla 16, Riberalta, Bolivia; 58 Universidad Autónoma del Beni, Campus Universitario, Riberalta, Bolivia; 59 Northern Arizona University, Flagstaff, AZ 86011, USA; 60 Department of Geography and the Environment, University of Texas at Austin, Austin, TX 78712, USA

**Keywords:** tropical tree, trait, convergent evolution, divergent selection, phylogenetic signal

## Abstract

Lineages tend to retain ecological characteristics of their ancestors through time. However, for some traits, selection during evolutionary history may have also played a role in determining trait values. To address the relative importance of these processes requires large-scale quantification of traits and evolutionary relationships among species. The Amazonian tree flora comprises a high diversity of angiosperm lineages and species with widely differing life-history characteristics, providing an excellent system to investigate the combined influences of evolutionary heritage and selection in determining trait variation. We used trait data related to the major axes of life-history variation among tropical trees (e.g. growth and mortality rates) from 577 inventory plots in closed-canopy forest, mapped onto a phylogenetic hypothesis spanning more than 300 genera including all major angiosperm clades to test for evolutionary constraints on traits. We found significant phylogenetic signal (PS) for all traits, consistent with evolutionarily related genera having more similar characteristics than expected by chance. Although there is also evidence for repeated evolution of pioneer and shade tolerant life-history strategies within independent lineages, the existence of significant PS allows clearer predictions of the links between evolutionary diversity, ecosystem function and the response of tropical forests to global change.

## 1. Introduction

Evolutionary heritage may act as a major constraint on the ecological roles that species in a lineage can occupy. Even under a random model of trait evolution where functional traits drift in state over time (e.g. a Brownian motion model), we would expect closely related species to have similar functional trait values and similar ecologies due to their shared common ancestry [1,2]. However, both divergent selection and convergent evolution lead to weaker relationships between species relatedness and their ecological similarity [1,3,4]. Hence, although it is often assumed that close relatives are more similar because they retain the ecological characteristics of their ancestors, in many clades the ancestral character state may not be conserved. Thus, rather than being simply assumed, the tendency of closely related species to have similar ecological characteristics needs to be tested.

The strength of the link between trait variation and phylogenetic relatedness has a wide range of implications for understanding ecological and evolutionary processes and can be measured by the magnitude of phylogenetic signal (PS) [1,2]. For example, if a selected trait has significant PS, the relatedness of species can help us to understand the underlying mechanisms that drive community structure [5–7]. The presence of significant PS also suggests that the sum of phylogenetic distances among species that occur within a community (i.e. phylogenetic diversity) is a useful proxy for functional diversity and that, in turn, phylogenies of tree taxa may contribute to understanding ecosystem function [8,9]. In addition, if trait values are more similar than expected by chance among closely related lineages, we can predict the trait values for species where trait data are not available.

Understanding the relative importance of evolutionary heritage versus selection in determining trait variation requires large-scale quantification of traits and evolutionary relationships among species. The Amazonian tree flora comprises a high diversity of angiosperm lineages and species with widely differing life-history characteristics, providing an excellent system to investigate these processes. Previous studies of the degree of PS among traits of tropical trees, such as seed mass, leaf structure and chemistry, trunk characteristics and range size, have shown variable results [6,7,10–13]. For example, some studies show significant PS [6,7,13], while for the same traits other studies have failed to detect any PS, with closely related species exhibiting rather different trait values [10,11]. A key limitation of many of these studies is the limited spatial and phylogenetic scale of study, as well as the resolution of the phylogeny that they have used [14]. Here, we explore patterns of PS at large spatial and phylogenetic scales using a sequence-based phylogeny to test whether there are significant levels of PS for four key traits related to the major axes of life-history variation among tropical trees: tree growth and mortality rates, wood density and potential tree size. These traits are related to resource acquisition and allocation, defence and dispersal ability [15,16] and represent important axes of functional variation which drive variation in plant performance and function in many ecosystems [17]. Moreover, those traits are strongly related to differences in carbon fluxes and storage among species [18]. As a result, understanding PS in these traits may help to understand and model ecosystem processes in such highly diverse tropical forests such as Amazonia, which may harbour more than 16 000 tree species [19].

Studying PS at large spatial scales is important because the scale of study affects the strength of PS. At small scales, patterns of PS can be obscured because co-occurring species represent just a small fraction of the species richness of clades [20,21]. Small spatial scales encompass limited environmental variation, so the species pool is limited to representatives of different lineages that may have similar ecological traits and environmental requirements: this pattern results in a smaller range in traits and low PS. The strength of this effect depends on how environmental variability changes with spatial scale, on the degree of habitat specialization by species and the proportion of clades that are sampled in small-scale studies [6,7]. However, in general, larger spatial scales incorporate greater environmental heterogeneity and encompass a larger number of lineages with a wider range of trait values. Inferring patterns of PS that are more representative of evolutionary trends therefore typically requires measurement across large spatial scales, including a wide range of environmental conditions and taxa from a broad array of clades [22].

The patterns of PS also depend on traits under investigation and their specific evolutionary history. Some traits may exhibit phylogenetic conservatism where traits in specific lineages are constrained to certain trait values. For example, complex traits, such as growth and mortality, may depend in complex ways on multiple, interacting gene loci [23,24] which impose strong constraints on trait variation. Alternatively, traits may show no PS because they are under strong selective pressure and/or because they show phenotypic plasticity in response to environmental conditions [20,25].

Here, we use a large dataset of several hundred permanent forest plots that occur across a wide range of the environmental conditions from all nine Amazonian countries [26], to quantify key demographic traits of more than 300 lineages of tropical trees and explore the PS of these traits using recently published molecular genus- [13] and species-level phylogenies ([27], KG Dexter & RT Pennington 2013, unpublished data). By exploring how traits are correlated and the strength of PS, our goal is to address the fundamental question of whether repeated convergent and divergent evolution of life-history strategies has erased PS for life-history-related traits in tropical trees, or whether phylogenetic information can be used to understand ecosystem function in the world's most diverse and ecologically important forest.

## 2. Material and methods

### (a) Plot data

**Figure 1.**
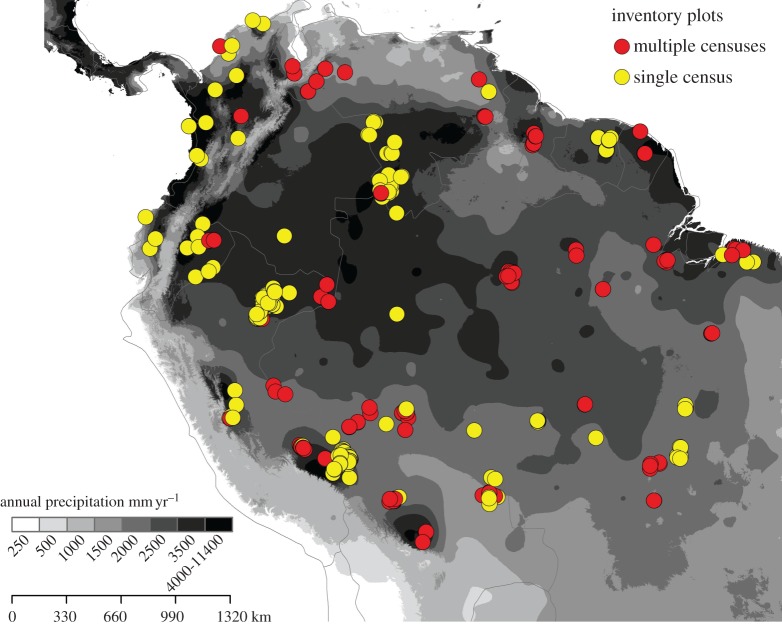
Map of location of 577 selected plots in lowland tropical South America over a backcloth of the precipitation gradient (annual precipitation, from the WorldClim dataset [28]). The map shows plots, with annual precipitation greater than 1300 mm yr^−1^ and altitude less than 500 m. Yellow circles: single census, plots used exclusively for estimating wood density and potential tree size; red circles: multi censuses, plots used for estimating wood density, potential tree size, growth and mortality rates.

This study used inventory data from all trees and palms greater than or equal to 10 cm diameter (DBH) in 577 forest plots from the RAINFOR forest plot network (figure 1; electronic supplementary material, S1) across lowland closed-canopy South American tropical forests. This network is centred on Amazonia and includes plots in forests on the Guiana Shield, in the Choco and northern South America; however, hereafter for simplicity we refer to this sampling region as ‘Amazonia’. Plots are located in old growth, unlogged forests and range in size from 0.04 to 25 ha (most being 1 ha). They span a precipitation gradient from 1300 to 7436 mm yr^−1^ [28], a broad range of soil types [29], and are found below 500 m in elevation. Data were extracted from the ForestPlots.net database which curates tree-by-tree records from RAINFOR and other plot networks [26,30].

For productivity and mortality analyses, we used a subset of 257 repeated census plots with a minimum monitoring period of 2 years from 1962 to 2014. Mean census interval length is 4.4 years and plot mean total monitoring period is 9.9 years. During each census, all surviving trees and palms were measured, dead trees were documented and new trees with greater than or equal to 10 cm DBH were recorded. More detailed measurement methods and plot characteristics have been previously published (e.g. [31,32]). All recorded species and genus names were checked and standardized using the Taxonomic Name Resolution Service [33]. We excluded all trees and palms not identified to genus level (7.9% of stems).

### (b) Trait data

Trait mean values of potential tree size, mean and maximum growth rates, mortality rates and wood density were calculated at both the genus and species levels. Our main analyses were performed at the genus level and covered genera present in a recently published genus-level phylogeny for Amazonian trees [13]. Species-level trait data for those clades where we had species-level phylogenies with sufficient sampling of species in our dataset (more than 20 species): Burseraceae [27] and *Inga* (KG Dexter & RT Pennington 2013, unpublished data) were used to investigate whether patterns of PS at the genus level were consistent with species-level patterns. Species-level trait data were also used to account for intrageneric variation in the genus-level analyses of PS: the species-level data were used to calculate the standard error of each trait within each genus, and these values were incorporated into the calculations of PS (described below) [34]. In the methods below, all the details are given for trait values calculated at the genus level; similar calculations and methods were used at the species level.

Potential tree size, mean and maximum growth rates were all calculated in terms of tree diameter, basal area and biomass for each genus with at least 20 individuals across multiple censuses.

Potential tree size was estimated as the 95th percentile of the size distribution of all trees within each genus. For trees with multiple measurements, we selected the maximum size across different censuses to define these distributions. Tree above-ground biomass per stem was calculated using the pan-tropical, three parameter allometric equation (diameter, wood density and *E*) of Chave, Rejou-Mechain [35], which assumes that tree diameter–height relationships depend linearly on bioclimatic variables (*E*), where *E* is a measurement of environmental stress based on measures of temperature seasonality and precipitation seasonality derived from the WorldClim dataset [28] and a measure of Climatic Water Deficit extracted from a global gridded dataset [35]. Palm biomass was estimated using a palm-specific allometric equation based on diameter [36].

For each genus, we computed both mean growth rate and the 95th percentile of growth rates, to represent maximum growth rates within each genus, across all stems. To calculate these parameters, mean stem-level growth rate was first estimated as the mean growth per year across multiple censuses and maximum stem-level growth as the maximum growth rate per year calculated across multiple censuses. Trees with mean negative growth rates (0.9% of stems) were excluded in order to normalize the data (similar to [37]). We also excluded palms, which do not have secondary growth, nine trees exhibiting diameter growth greater than 80 mm yr^−1^ which may represent recording errors and stems where diameter measurements were not made using a tape measure (0.12% of all stems). If a change in the point of measurement (POM) was made during the measurement record of any given tree, we calculated growth rates using the arithmetic mean of the diameter measured at the original POM and the diameter at the new POM [38].

Mortality rates were estimated for all genera with a minimum of 100 individuals in the plot data, based on the number of individuals found alive in the initial and final censuses of each plot. To estimate average mortality rates within each genus, the survival probability of individual trees within each clade was modelled as an exponentially declining function of the monitoring period while accounting for variation in tree size [39,40].

To account for the wide range of environmental conditions across plots [29], we used mixed models to calculate genus-level values of potential tree size, mean and maximum growth rates and mortality rates while accounting for systematic variation in these parameters among plots [40] (see the electronic supplementary material, S2).

Wood density data were extracted from the Global Wood Density database [41,42] and average values calculated for each genus in the phylogeny [43].

### (c) Trait correlations

To identify relationships among genus-level traits, we conducted a phylogenetic principal component analysis (PPCA) [44] including genera where we have a complete set of trait data. PPCA incorporates the expected correlation among traits due to their shared evolutionary history into the principal component analyses [45]. We standardized trait values to a mean of zero and unit variance to ensure that each trait contributed equally to the PPCA.

### (d) Phylogenetic signal

In order to estimate PS for traits, we used Blomberg's *K* [1]. This metric quantifies the amount of variance in an observed trait in relation to the expected trait variance under a Brownian motion model of evolution [1,4]. Under this model of evolution, trait values drift randomly over time, with small changes being more likely than large changes within a given unit of time (trait values at *t*_1_ are chosen from a normal distribution centred on the trait value at *t*_0_). This model generates trait data where the covariance among trait values for taxa is proportional to the duration of their shared evolutionary history [4]. Values of *K* equal to 0 indicate that there is no PS, while *K* equal to 1 indicates high PS and is the expected value under a Brownian motion model of evolution. Intermediate values (0 < *K* < 1) indicate intermediate levels of PS. To assess significance in *K*, we recalculated *K* on the tree with randomized tips a thousand times, and compared the simulated values with the observed value of *K*. If the observed value fell outside the range given by 2.5–97.5 percentiles of the simulated values, this value was considered significant.

We accounted for intrageneric trait variation in the calculation of *K* by measuring the standard error for each genus, treating individual genera as species and intrageneric variation as intraspecific variation *sensu* [34]. For genera where the standard error could not be computed, we assigned the mean value of the standard error for all genera with estimates for multiple species [34]. Including this within-genus variation allows us to account for uncertainty in trait estimation (e.g. population variation and measurement error), improve parameter estimation and reduce bias in the calculation of PS [1,34].

We also calculated PS using Pagel's *λ* [46] in order to explore whether our results were dependent on the particular method used to calculate PS (see the electronic supplementary material, S3).

### (e) Sensitivity analysis

To investigate whether our results were affected by the spatial scale of our study, we repeated our analyses using 26 plots within 55 km of each other near Manaus. Similarly, to verify whether our results were affected by our use of genus-level data, we conducted the same analyses at the species level for the genus *Inga* and the Protieae (Burseraceae). Likewise, to investigate whether the number of lineages included in the analyses affected the extent of PS, we repeated the calculations of PS with just the genera with a complete set of trait values (214).

Statistical analyses were performed in the R v. 3.1.1 program [47], using ape [48], phytools [44] and data.table [49] packages.

## 3. Results

### (a) Trait data

**Figure 2.**
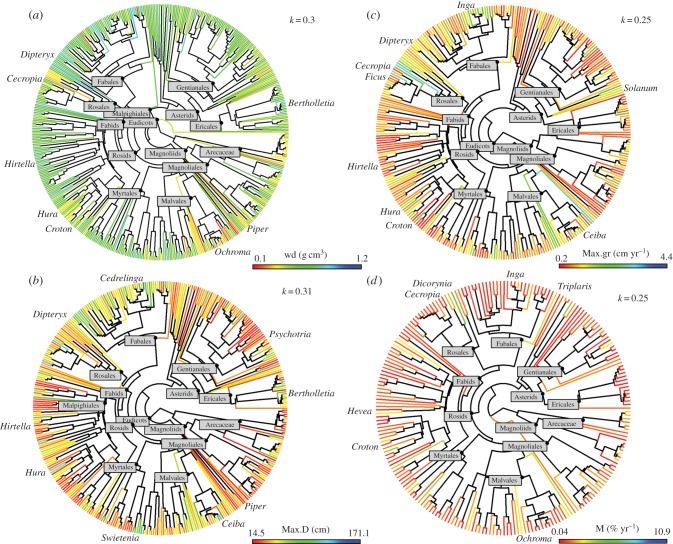
Phylogeny (based on rbcL and matK plastid gene) of 497 Amazonian tree and palm genera. Number of genera varied in the different phylogenies according to the selection criterion for each trait (see Material and methods). Branches are coloured according to (*a*) wood density (wd g.cm^3^), (*b*) potential tree size in diameter (Max.D cm), (*c*) maximum tree growth in diameter (Max.gr cm yr^−1^) and (*d*) mortality rates (% yr^−1^). Continuous traits were coloured using a continuous colour gradient, with colour codes indicate the wide range of trait values, from blue to red, indicating higher and lower trait values, respectively. Phylogenies for each trait with all tips labelled are available in the electronic supplementary material (S6).

All traits measured varied substantially among genera (table 1 and figure 2): wood density varied eightfold, potential size in tree diameter 12-fold, potential size in biomass 814-fold, maximum growth rates in tree diameter 23-fold, mean diameter growth rates 35-fold and mortality rates 275-fold. Overall, the trait values after correcting for environmental variation and those estimated directly from the database without accounting for variation among plots were highly correlated with each other (*p* < 0.001 in all cases and *τ* ranging from 0.59 to 0.79).

**Table 1.** Summary of trait data, including number of genera per trait, number of species and number of individuals used for selection criterion, minimum, maximum and mean trait values per genera. In addition, PS for absolute trait values, accounting for intrageneric variation, environmental variation and both environmental and intrageneric variation. PS measured using Blomberg's *K*. Statistical significance in probability tests are indicated by asterisks.

	traits	units	no. ind	no. genera	no. species	range	mean	PS (*K*)			
								intrageneric variation			
								no	yes	no	yes
								environmental variation			
								no	no	yes	yes
wood density	wd	g.cm^3^	—	497	1324	0.15–1.21	0.61	0.26***	0.30***	—	—
potential size	maximum diameter	cm	244 362	383	1412	14.5–171.1	459.4	0.23***	0.31***	0.20***	0.29***
	maximum diameter × wd	—	244 362	383	1412	4.94–154.69	28.08	0.27***	0.34***	0.25***	0.32***
	maximum basal area	m^2^	244 362	383	1412	0.02–2.3	0.21	0.23***	0.31***	0.21**	0.26***
	maximum basal area × wd	—	244 362	383	1412	0.01–0.13	0.13	0.26***	0.32***	0.23***	0.29***
	maximum biomass	kg	244 362	383	1412	54.63–44443.1	2760.6	0.25***	0.28***	0.22***	0.28***
growth rates	maximum growth in diameter	cm yr^−1^	134 303	329	1024	0.19–4.38	0.93	0.19***	0.25***	0.18***	0.25***
	maximum growth in basal area	m^2^ yr^−1^	134 303	329	1024	0.003–0.03	0.005	0.22***	0.32***	0.21***	0.29***
	maximum growth in biomass	kg yr^−1^	134 303	329	1024	0.21–95.23	6.17	0.25***	0.39***	0.23***	0.33***
	mean growth in diameter	cm yr^−1^	133 656	327	1000	0.05–1.74	0.26	0.18***	0.25***	0.19***	0.29***
	mean growth in basal area	m^2^ yr^−1^	133 656	327	1000	0–0.01	0	0.20***	0.27***	0.19***	0.29***
	mean growth in biomass	kg yr^−1^	133 656	327	1000	0.15–21.76	1.67	0.23***	0.30***	0.19***	0.25***
mortality	mean stem mortality	% yr^−1^	156 495	221	306	0.04–10.98	1.08	0.17**	0.25**	—	—
PPCA1	—		—	214	—	—	—	0.18**	—	—	—
PPCA2	—		—	214	—	—	—	0.21***	—	—	—

***p < 0.001, **p < 0.05, *p < 0.1.

### (b) Trait relationships

**Figure 3.**
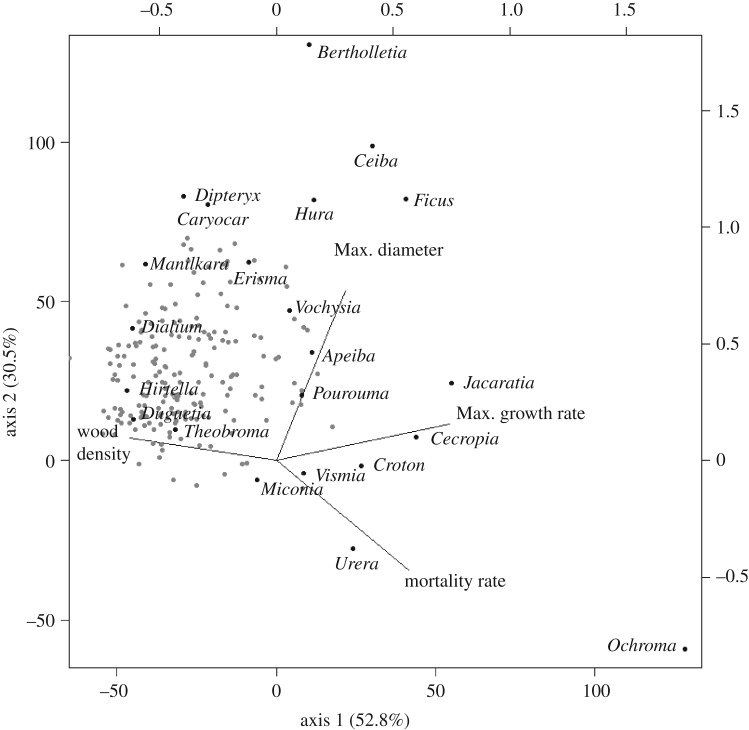
PPCAs for the first two principal components with PC loadings for the four traits studied here: wood density, potential tree size in terms of diameter (Max. diameter), potential growth rates in terms of diameter (Maxgr. growth rate) and annual mortality rates (Mortality rate). Points represent 221 genera of trees; position of 22 key genera marked in bold and named.

Trait associations among lineages were analysed with a PPCA: 83% of the variation in the four-dimensional space was accounted for by the first two axes (figure 3). The first axis (PPCA1) explained 52.8% of the variation and shows strong positive loadings for mortality and maximum growth rates, while wood density was negatively associated with this axis (electronic supplementary material, S4). PPCA1 thus represents a continuum from pioneer and light demanding lineages with low wood density and fast demographic traits (e.g. high mortality and growth rates) to non-pioneer lineages with high wood density and slow demographic rates. The second axis (PPCA2) explained 30.5% of the variation and was associated more closely with potential tree size, and reflects the variation from individuals of understory genera, to individuals of canopy and emergent lineages (figure 3).

### (c) Phylogenetic signal

All traits and the first two PPCA axes exhibited significant PS, with closely related genera being more similar than expected by chance, using either Bloomberg's *K* (table 1) or Pagel's *λ* (electronic supplementary material, S3). Because estimates of Pagel's *λ* and Blomberg's *K* are strongly correlated and most studies of PS in tropical trees have focused on the *K* metric rather than *λ*, we focus our results and discussion on the calculations using Blomberg's *K-*value.

Traits showed significant and similar values for *K*, varying from 0.25 to 0.39 and from 0.17 to 0.27, with and without accounting for intrageneric variation, respectively. These *K*-values indicate that evolutionarily related genera tend to be more similar to each other, but less than expected under a BM model of evolution (table 1). Finally, removing the environmental contribution to trait variation did not substantially alter the magnitude of PS (table 1).

### (d) Sensitivity analyses

Although using just the Manaus plot data significantly reduced the number of genera, species and individual trees included in the analyses, PS at smaller spatial scales showed similar patterns to PS calculated using the whole dataset (electronic supplementary material, S5). Similarly, reducing the number of lineages to genera we had all trait values showed congruent patterns of PS (electronic supplementary material, S5). In addition, all traits showed similar or slightly higher Blomberg's *K* values for just *Inga* or Protieae than for all taxa together (electronic supplementary material, S5).

## 4. Discussion

This is the first study, to our knowledge, to investigate the extent of PS for traits that quantify the main axes of life-history variation in survival and growth of trees at such a large phylogenetic and spatial scale. Our results demonstrate that for Amazonian forests, closely related genera have similar life-history strategies, with all traits showing similar levels of PS (table 1 and figure 2; electronic supplementary material, S6). The similar level of PS found across all the different, correlated traits suggests that the main axes of life-history variation among lineages of Amazonian trees may represent the result of repeated evolution of a suite of coordinated functional characteristics.

### (a) Relationships among traits

Strong correlations among traits were represented by two major axes of variation, which are likely to be associated with adaptations to horizontal and vertical light gradients. Ecological differences among species adapted to gaps versus the shaded understory or to the understory versus the canopy are well established as the principal axes of functional variation among tropical forest tree species [50,51]. The first axis runs from pioneer and light demanding genera with low wood density and fast demographic traits (e.g. high mortality and high growth rates) to shade tolerant genera with dense wood and slow demographic traits. The second axis represents variation in tree size and contrasts understorey genera, from lineages of canopy trees. For example, these axes distinguish *Cecropia* and *Croton*, classic pioneers with low wood density and fast demographic traits, from *Hirtella*—a typically dense-wooded and slow-growing understory genus of trees. Lineages of emergent trees which all achieve very large potential tree sizes (e.g. *Bertholletia*, *Ceiba*, *Hura, Dipteryx*), are also distinguished in this analysis by their different wood densities and growth rates (figure 3).

### (b) Phylogenetic signal

Our results demonstrate significant levels of PS among demographic and structural traits of tropical trees, with Blomberg's *K* ranging from 0.25 to 0.39. This pattern suggests that evolutionary relationships provide useful information about the ecological similarity of these lineages. However, while our analyses of PS shows that evolutionarily related lineages have more similar traits than expected by chance, their values are lower than expected under a pure BM model of evolution (table 1 and figure 2) under which *K*-values would be close to 1. PS can be lower than expected under BM if there is convergent evolution across distantly related lineages and/or divergent selection among closely related groups [3,4]. This result therefore suggests that there has been repeated convergent evolution and/or divergent selection, along the two main axes of variation identified by the PPCA analysis (figure 3). This finding suggests that adaptations to light gaps, or understorey and canopy light environments, have repeatedly evolved within multiple lineages of tropical trees as shown by the different pioneer and shade tolerant genera within a series of unrelated families (e.g. *Cecropia* versus *Brosimum* (Urticaceae/Moraceae), *Vismia* versus *Calophyllum* (Clusiaceae) and *Inga* versus *Dipteryx*/*Parkia* (Fabaceae); figure 2).

### (c) Sensitivity analyses

The PS found here for trees across lowland closed-canopy South American forests is generally stronger than previously reported in the literature for tropical forests in smaller scale analyses (electronic supplementary material, S7). In previous studies, some traits showed low but significant PS [6,7,13], while others have even found that traits are randomly dispersed over the phylogeny [10,11]. However, although *K*-values are standardized to allow comparison between traits and phylogenetic trees [1,4], direct comparisons of PS are affected by differences in the spatial and taxonomic scale of the studies, the number of lineages and the use of different kinds of phylogenies.

A first issue for comparing the extent of PS among studies is variation in spatial scales. However, here we show that the higher PS in this study is unlikely to be an artefact of our larger spatial scale: restricting our analyses to 26 plots around Manaus shows consistent patterns, with similar levels of PS for all traits compared to analyses for the whole Amazon (electronic supplementary material, S5).

Secondly, different numbers of lineages in different studies may play a role in determining variation in the extent of PS. Although Blomberg's *K* is efficient at detecting the strength of similarity among closely related lineages for sample sizes greater than 20 [1], the ability to detect different levels of PS may increase with larger sample sizes [52]. To address this issue, we conducted a set of analyses restricted to genera for which we had all trait values (214 genera). As estimates of *K* are highly consistent when we include fewer genera (electronic supplementary material, S5), it appears that the number of lineages is unlikely to have caused the observed trends of high levels of PS for our traits.

Thirdly, most of previous studies [6,7,10–12] were conducted at the species level, and taxonomic scale can also affect the degree of PS. PS in any trait may vary at different taxonomic scales; a single trait can have high similarity at one level (e.g. genus level) but this pattern can break down at higher or lower taxonomic levels [52]. Here, the PS of these traits at the species level within the Protieae and *Inga* were similar or slightly greater than for the genus-level results (electronic supplementary material, S5), suggesting that our results are consistent at finer taxonomic levels. However, as our analyses at low taxonomic levels were limited to two lineages it remains to be fully tested whether the result indeed holds within all clades of Neotropical trees.

Finally, the use of different kinds of phylogenies is likely to affect the extent of similarity among related species that is reported in different studies (electronic supplementary material, S7). Much previous work was carried out using community-level phylogenies, restricted to locally co-occurring species [6,12] and in many cases using unresolved phylogenies with relationships represented as polytomies [11]. Such community-level phylogenies may lack sister lineages for many clades that may be critical to effectively measure PS. In addition, the use of trees with many polytomies, e.g. those which add genera and species as polytomies onto backbone family-level trees [53], leads to uncertainty in PS estimates [14]. More importantly, phylogenetic sampling may play a major role in determining the extent of PS. Although the genus-level phylogeny used here is far from complete, our analyses do encompass a far wider range of lineages than previous studies, including the major angiosperm lineages present in the Amazon basin.

Our results demonstrate that there is significant PS for key demographic and structural traits in tropical forests. This finding opens the way for clearer predictions of how evolutionary diversity relates to ecosystem structure and function, and how different drivers will, in turn, affect the evolutionary diversity of Amazonian forests. For example, this study suggests that community-level measures of evolutionary relatedness among species are likely to be good predictors of the structure and functioning of these ecosystems [8,9]. These results also indicate that changes in environmental conditions or disturbance regimes that favour particular life-history strategies will ultimately erode evolutionary diversity [54,55], although the presence of some convergent evolution across lineages may prevent significant loss of phylogenetic diversity over some scales of anthropogenic disturbance [56]. Our results may therefore help to resolve why different studies of the effect of disturbance on phylogenetic diversity have obtained contrasting results [54–56]: in particular, this study suggests that investigating the PS of traits that influence species ability to persist after disturbance within the species pool of interest will be critical to understand how disturbance will alter phylogenetic diversity. Finally, our results also suggest that any long-term changes in the evolutionary diversity of intact Amazonian forests may indicate functional shifts in these diverse ecosystems. Overall, the phylogenetic structure of life-history strategies within Amazon tree communities described in this study helps to provide a predictive framework to understand how such complex systems will respond to global change and anthropogenic disturbance.

## Supplementary Material

List of plot data used in the present study

## Supplementary Material

Methods for calculating trait intrinsic value

## Supplementary Material

Supporting reults

## Supplementary Material

Phylogeny of 497 Amazonian tree genera

## Supplementary Material

Comparison between published values of phylogenetic signal and values found in the present study

## Data accessibility

The permanently archived data package of the genus-level trait dataset (wood density, potential tree size, maximum and mean growth rates and mortality rates) are available from https://doi.org/10.5521/FORESTPLOTS.NET/2016_4.
