# Supplementary material for: Evolutionary heritage influences Amazon tree ecology
Source: Proc Biol Sci. 2016 Dec 14;283(1844):20161587. doi: 10.1098/rspb.2016.1587 (PMC5204144; doi:10.1098/rspb.2016.1587)
Supplement: List of plot data used in the present study [file rspb20161587supp1.pdf]

Proceedings of the Royal Society B

SUPPORTING INFORMATION

Evolutionary heritage influences Amazon tree ecology

Fernanda Coelho de Souza, Kyle G. Dexter, Oliver L. Phillips, Roel J.W. Brienen, Jerome Chave, David R. Galbraith, Gabriela Lopez-Gonzalez, Abel Monteagudo-Mendoza, R. Toby Pennington, Lourens Poorter, Miguel Alexiades, Esteban Álvarez-Dávila, Ana Andrade, Luis E.O.C. Aragão, Alejandro Araujo-Murakami, Eric J.M.M. Arets, Gerardo A. Aymard C., Christopher Baraloto, Jorcely Barroso, Damien Bonal, Rene G.A. Boot, José L.C. Camargo, James A. Comiskey, Fernando Cornejo Valverde, Plínio B. de Camargo, Anthony Di Fiore, Fernando Elias, Terry L. Erwin, Ted R. Feldpausch, Leandro Ferreira, Nykolas M.F. Fyllas, Emanuel Gloor, Bruno Herault, Rafael Herrera, Niro Higuchi, Eurídice N. Honorio Coronado, Timothy J. Killeen, William F. Laurance, Susan Laurance, Jon Lloyd, Thomas E. Lovejoy, Yadvinder Malhi, Leandro Maracahipes, Beatriz S. Marimon, Ben H. Marimon-Junior, Casimiro Mendoza, Paulo Morandi, David A. Neill, Percy Núñez Vargas, Edmar A. Oliveira, Eddie L. Oliveira, Walter A. Palacios, Maria C. Peñuela-Mora, John J. Pipoly III, Nigel C.A. Pitman, Adriana Prieto, Carlos A. Quesada, Hirma Ramirez-Angulo, Agustin Rudas, Kalle Ruokolainen, Rafael P. Salomão, Marcos Silveira, Juliana Stropp, Hans ter Steege, Raquel Thomas-Caesar, Peter van der Hout, Geertje M.F. van der Heijden, Peter J. van der Meer, Rodolfo V. Vasquez, Simone A. Vieira, Emilio Vilanova, Vincent A. Vos, Ophelia Wang, Kenneth R. Young, Roderick J. Zagt, Timothy R. Baker

Doi: 10.1098/rspb. 2016.1587

Additional Supporting information S1

Table 1. List of plots encompassing the Floristic tree inventories for 577 plots compiled from RAINFOR database, with their respective coordinates in Latitude (Lat.) and Longitude (Long.) , area in hectare, number of individuals with diameter equal or superior to 10 cm, number of species, number of genera and data contributors. Plots with long term data and minimum of 2 years census interval (257) are assigned.

Plot Code	Lat.	Long.	Plot size ha	Nº Ind.	Nº Species	Nº Genera	Multiple census	Data contributors
AGJ-01	-11.89	-71.36	2	1637	186	120	X	John Terborgh
AGP-01	-3.72	-70.31	1	826	291	143	X	Oliver Phillips; Agustin Rudas; Alvaro Cogollo; Esteban Alvarez; Adriana Prieto; Jon Lloyd
AGP-02	-3.72	-70.3	1	877	292	149	X	Oliver Phillips; Agustin Rudas; Alvaro Cogollo; Esteban Alvarez; Adriana Prieto; Jon Lloyd
ALF-01	-9.6	-55.94	1	569	143	91	X	Ted Feldpausch; Beatriz Marimon; Ben Hur Marimon; Jon Lloyd
ALF-02	-9.58	-55.92	1	584	108	77	X	Ted Feldpausch; Beatriz Marimon; Ben Hur Marimon; Jon Lloyd
ALM-01	-11.8	-71.47	2	1754	329	180	X	John Terborgh; Roel Brienen; Nigel Pitman; Fernando Cornejo
ALP-01	-3.95	-73.43	1	752	323	152	X	Abel Monteagudo; Tim Baker; Oliver Phillips; Roel Brienen; Yadvinder Malhi; Rodolfo Vasquez

Plot Code	Lat.	Long.	Plot size ha	Nº Ind.	Nº Species	Nº Genera	Multiple census	Data contributors
ALP-02	-3.95	-73.44	1	792	333	162	X	Abel Monteagudo; Tim Baker; Oliver Phillips ;Roel Brienen
ALP-05	-3.96	-73.44	0.1	65	50	39	-	Oliver Phillips
ALP-06	-3.95	-73.44	0.1	64	56	44	-	Oliver Phillips
ALP-10	-3.95	-73.41	0.1	93	73	59	-	Oliver Phillips; Tim Baker; Roel Brienen; Rodolfo Vasquez; Abel Monteagudo
ALP-16	-3.94	-73.43	0.1	86	66	55	-	Oliver Phillips; Tim Baker; Roel Brienen; Rodolfo Vasquez; Abel Monteagudo
ALP-17	-3.94	-73.43	0.1	106	46	42	-	Oliver Phillips; Tim Baker; Roel Brienen; Rodolfo Vasquez; Abel Monteagudo
ALP-18	-3.95	-73.43	0.1	75	42	37	-	Oliver Phillips; Tim Baker; Roel Brienen; Rodolfo Vasquez; Abel Monteagudo
ALP-19	-3.96	-73.44	0.1	94	65	55	-	Oliver Phillips; Tim Baker; Roel Brienen; Rodolfo Vasquez; Abel Monteagudo
ALP-20	-3.96	-73.43	0.1	63	48	36	-	Oliver Phillips; Tim Baker; Roel Brienen; Rodolfo Vasquez; Abel Monteagudo
ALP-23	-3.95	-73.42	0.1	63	38	34	-	Oliver Phillips; Rodolfo Vasquez
ALP-24	-3.96	-73.43	0.1	44	27	25	-	Oliver Phillips; Rodolfo Vasquez
ALP-25	-3.95	-73.44	0.1	46	31	24	-	Oliver Phillips; Rodolfo Vasquez
ALP-26	-3.95	-73.41	0.1	65	14	11	-	Oliver Phillips; Rodolfo Vasquez
ALP-30	-3.95	-73.43	1	560	112	71	X	Oliver Phillips; Tim Baker; Roel Brienen; Rodolfo Vasquez; Abel Monteagudo
ALP-40	-3.94	-73.44	1	1270	43	33	X	Oliver Phillips; Roel Brienen; Rodolfo Vasquez; Abel Monteagudo; Freddy Ramirez Arevalo
AMA-02	5.58	-77.5	1	472	106	79	X	Esteban Álvarez Dávila; Oliver Phillips
AMD-01	-1.83	-46.75	1	536	138	86	-	Ima Vieira
AMD-02	-1.83	-46.75	1	453	121	77	-	Ima Vieira
AMI-01	-13.58	-68.76	0.1	81	54	49	-	Alwyn Gentry; Percy Núñez Vargas
AMI-02	-13.58	-68.76	0.1	85	47	44	-	Alwyn Gentry
ANC-01	3.77	-76.87	0.1	96	34	32	-	Alwyn Gentry
ARC-10	-0.41	-72.33	0.1	89	42	37	-	Alwyn Gentry
ARC-11	-0.41	-72.31	0.04	66	16	15	-	Alwyn Gentry
BAC-01	7.46	-71.01	0.25	133	44	42	X	Emilio Vilanova; Hirma Ramirez-Angulo; Armando Torres-Lezama ; Geertje van der Heijden; Oliver Phillips
BAC-02	7.46	-71.01	0.25	124	34	32	X	Emilio Vilanova; Hirma Ramirez-Angulo; Armando Torres-Lezama ; Geertje van der Heijden; Oliver Phillips
BAC-03	7.46	-71.01	0.25	91	22	21	X	Emilio Vilanova; Hirma Ramirez-Angulo; Armando Torres-Lezama ; Geertje van der Heijden; Oliver Phillips
BAC-04	7.46	-71.01	0.25	108	29	29	X	Emilio Vilanova; Hirma Ramirez-Angulo; Armando Torres-Lezama ; Geertje van der Heijden; Oliver Phillips

Plot Code	Lat.	Long.	Plot size ha	Nº Ind.	Nº Species	Nº Genera	Multiple census	Data contributors
BAC-05	7.47	-71.02	0.25	135	33	32	X	Emilio Vilanova; Hirma Ramirez-Angulo; Armando Torres-Lezama ; Geertje van der Heijden; Oliver Phillips
BAC-06	7.47	-71.02	0.25	87	30	29	X	Emilio Vilanova; Hirma Ramirez-Angulo; Armando Torres-Lezama ; Geertje van der Heijden; Oliver Phillips
BAR-01	-11.9	-71.42	1	816	209	124	X	Abel Monteagudo; John Terborgh; Oliver Phillips; Percy Núñez Vargas
BBS-01	4.93	-55.22	1	635	123	91	-	Hans ter Steege
BBS-02	4.93	-55.19	1	569	113	86	-	Hans ter Steege
BBS-03	4.95	-55.19	1	630	104	82	-	Hans ter Steege
BBS-04	4.97	-55.18	1	465	98	81	-	Hans ter Steege
BBS-05	4.99	-55.2	1	515	112	92	-	Hans ter Steege
BBS-06	4.94	-55.18	1	544	106	83	-	Hans ter Steege
BBS-07	4.92	-55.13	1	458	104	87	-	Hans ter Steege
BBS-08	4.93	-55.14	1	555	97	77	-	Hans ter Steege
BBS-09	4.95	-55.19	1	622	91	75	-	Hans ter Steege
BCA-01	3.97	-77.07	0.1	94	65	54	-	Emilio Vilanova
BCU-01	11.14	-73.47	0.1	63	26	26	-	Emilio Vilanova
BDF-01	-2.34	-60.1	2	1689	383	155	X	William Laurence; Susan Laurance; Ana Andrade; Jose Camargo, Thomas Lovejoy
BDF-03	-2.42	-59.85	1	793	298	137	X	William Laurence; Susan Laurance; Ana Andrade; Jose Camargo, Thomas Lovejoy
BDF-04	-2.43	-59.85	1	934	316	140	X	William Laurence; Susan Laurance; Ana Andrade; Jose Camargo, Thomas Lovejoy
BDF-05	-2.43	-59.85	1	848	296	111	X	William Laurence; Susan Laurance; Ana Andrade; Jose Camargo, Thomas Lovejoy
BDF-06	-2.41	-59.86	3	2549	487	181	X	William Laurence; Susan Laurance; Ana Andrade; Jose Camargo, Thomas Lovejoy
BDF-07	-2.4	-59.9	1	862	302	132	X	William Laurence; Susan Laurance; Ana Andrade; Jose Camargo, Thomas Lovejoy
BDF-08	-2.4	-59.9	1	831	264	116	X	William Laurence; Susan Laurance; Ana Andrade; Jose Camargo, Thomas Lovejoy
BDF-09	-2.4	-59.85	1	759	290	130	X	William Laurence; Susan Laurance; Ana Andrade; Jose Camargo, Thomas Lovejoy
BDF-10	-2.39	-59.86	2	1670	461	178	X	William Laurence; Susan Laurance; Ana Andrade; Jose Camargo, Thomas Lovejoy
BDF-11	-2.38	-59.85	3	2203	505	192	X	William Laurence; Susan Laurance; Ana Andrade; Jose Camargo, Thomas Lovejoy
BDF-12	-2.39	-59.85	2	1492	438	176	X	William Laurence; Susan Laurance; Ana Andrade; Jose Camargo, Thomas Lovejoy
BDF-13	-2.4	-59.91	9	6527	637	220	X	William Laurence; Susan Laurance; Ana Andrade; Jose Camargo, Thomas Lovejoy
BDF-14	-2.36	-59.97	1	827	296	140	X	William Laurence; Susan Laurance; Ana Andrade; Jose Camargo, Thomas Lovejoy
BEE-01	-16.53	-64.58	1	669	93	70	X	Luzmila Arroyo;Alejandro Murakami-Araujo; Oliver Phillips
BEE-05	-16.53	-64.58	1	618	82	67	X	Luzmila Arroyo;Alejandro Murakami-Araujo; Oliver Phillips
BLS-01	0.61	-79.86	0.1	76	34	31	-	Alwyn Gentry
BNT-01	-2.64	-60.16	1	720	153	106	X	Niro Higuchi

Plot Code	Lat.	Long.	Plot size ha	Nº Ind.	Nº Species	Nº Genera	Multiple census	Data contributors
BNT-02	-2.64	-60.15	1	815	154	110	X	Niro Higuchi
BNT-04	-2.63	-60.15	1	785	154	113	X	Niro Higuchi
BNT-05	-2.63	-60.17	1	632	135	98	X	Niro Higuchi
BNT-06	-2.63	-60.17	1	622	131	96	X	Niro Higuchi
BNT-07	-2.63	-60.17	1	697	143	103	X	Niro Higuchi
BOG-01	-0.7	-76.48	1	743	242	141	X	Abel Monteagudo; Roel Brienen; Tony DiFiore; Nigel Pitman; Oliver Phillips
BOG-02	-0.7	-76.47	1	930	272	153	X	Abel Monteagudo; Roel Brienen; Tony DiFiore; Nigel Pitman; Oliver Phillips
BRP-01	2.83	-65.9	0.1	96	37	34	-	Gerardo Aymard
BVA-01	-4.24	-73.2	0.5	435	39	36	-	Eurídice Honorio; Tim Baker
CAG-01	-12.04	-69.11	0.1	53	31	28	-	Abel Monteagudo; Oliver Phillips; Fernando Valverde
CAG-02	-12.04	-69.1	0.1	76	32	31	-	Abel Monteagudo; Oliver Phillips; Fernando Valverde
CAG-03	-12.03	-69.1	0.1	78	25	24	-	Abel Monteagudo; Oliver Phillips; Fernando Valverde
CAG-04	-12.03	-69.1	0.1	51	38	34	-	Abel Monteagudo; Oliver Phillips; Fernando Valverde
CAG-05	-12.11	-69.14	0.1	58	43	41	-	Abel Monteagudo; Oliver Phillips; Fernando Valverde
CAG-06	-12.1	-69.18	0.1	51	24	22	-	Abel Monteagudo; Oliver Phillips; Fernando Valverde
CAG-07	-12.17	-69.14	0.1	48	31	27	-	Abel Monteagudo; Oliver Phillips; Fernando Valverde
CAG-08	-12.18	-69.13	0.1	62	42	36	-	Abel Monteagudo; Oliver Phillips; Fernando Valverde
CAG-09	-12.13	-69.11	0.1	54	33	33	-	Abel Monteagudo; Oliver Phillips; Fernando Valverde
CAG-10	-12.17	-69.05	0.1	75	26	24	-	Abel Monteagudo; Oliver Phillips; Fernando Valverde
CAG-11	-12.18	-69.05	0.1	57	21	20	-	Abel Monteagudo; Oliver Phillips; Fernando Valverde
CAI-01	8.7	-70.07	0.25	204	40	38	X	Emilio Vilanova; Geertje van der Heijden; Oliver Phillips
CAI-02	8.7	-70.07	0.25	112	40	34	X	Emilio Vilanova; Oliver Phillips
CAI-04	8.7	-70.07	0.25	142	41	34	X	Emilio Vilanova; Geertje van der Heijden; Oliver Phillips
CAS-01	2.03	-66.47	1	629	105	72	-	Gerardo Aymard
CAS-02	2.32	-66.48	1	672	73	54	-	Gerardo Aymard
CAS-03	1.92	-66.62	1	594	69	53	-	Gerardo Aymard
CAT-02	8.44	-71.77	0.25	100	16	15	X	Jean-Pierre Veillon
CAT-03	8.44	-71.77	0.25	85	15	15	X	Jean-Pierre Veillon
CAX-01	-1.74	-51.46	1	588	190	115	X	Antonio Lola da Costa da Costa
CAX-02	-1.74	-51.46	1	622	172	98	X	Antonio Lola da Costa
CAX-06	-1.72	-51.46	1	469	131	83	X	Antonio Lola da Costa; Luis Aragao
CAX-08	-1.85	-51.47	1	632	90	78	X	Antonio Lola da Costa
CBC-01	2.35	-66.55	0.1	86	33	31	-	Gerardo Aymard

Plot Code	Lat.	Long.	Plot size ha	Nº Ind.	Nº Species	Nº Genera	Multiple census	Data contributors
CBO-10	1.95	-66.98	0.1	79	31	28	-	Gerardo Aymard
CBP-01	-12.39	-69.31	0.1	60	36	29	-	Oliver Phillips; Percy Núñez Vargas
CBP-02	-12.4	-69.33	0.1	62	41	35	-	Oliver Phillips; Percy Núñez Vargas
CBP-03	-12.42	-69.28	0.1	60	34	31	-	Oliver Phillips; Percy Núñez Vargas
CBP-04	-12.41	-69.32	0.1	57	44	37	-	Oliver Phillips; Percy Núñez Vargas
CBP-05	-12.39	-69.31	0.1	63	42	37	-	Oliver Phillips; Percy Núñez Vargas
CBP-06	-12.43	-69.28	0.1	62	26	25	-	Oliver Phillips; Percy Núñez Vargas
CBP-07	-12.42	-69.33	0.1	46	30	27	-	Oliver Phillips; Percy Núñez Vargas
CBP-08	-12.43	-69.29	0.1	65	38	33	-	Oliver Phillips; Percy Núñez Vargas
CBP-09	-12.43	-69.29	0.1	62	41	34	-	Oliver Phillips; Percy Núñez Vargas
CDK-01	2.92	-66.63	0.1	109	37	33	-	Gerardo Aymard
CDM-01	-10.33	-75.3	1	520	124	83	-	Alwyn Gentry
CDM-02	-10.24	-75.21	0.1	50	30	26	-	Alwyn Gentry
CDT-01	2.8	-65.95	0.1	109	37	34	-	Gerardo Aymard
CHO-01	-14.39	-61.15	1	810	118	89	X	Luzmila Arroyo: Timothy Killeen
CJC-04	-12.67	-69.11	0.1	52	31	29	-	Abel Monteagudo; Oliver Phillips; Percy Núñez Vargas
CJC-05	-12.66	-69.08	0.1	69	25	23	-	Abel Monteagudo; Oliver Phillips; Percy Núñez Vargas
CJC-06	-12.64	-69.1	0.1	103	42	37	-	Abel Monteagudo; Oliver Phillips; Percy Núñez Vargas
CJC-07	-12.68	-69.11	0.1	98	40	35	-	Abel Monteagudo; Oliver Phillips; Percy Núñez Vargas
CJC-08	-12.69	-69.12	0.1	90	33	29	-	Abel Monteagudo; Oliver Phillips; Percy Núñez Vargas
CJC-09	-12.68	-69.18	0.1	71	48	43	-	Abel Monteagudo; Oliver Phillips; Percy Núñez Vargas
CJC-10	-12.68	-69.18	0.1	79	7	6	-	Abel Monteagudo; Oliver Phillips; Percy Núñez Vargas
CLO-01	9.53	-75.35	0.1	60	33	30	-	Alwyn Gentry
CLS-01	-12.6	-69.02	0.1	62	37	34	-	Abel Monteagudo; Oliver Phillips; Percy Núñez Vargas
CLS-02	-12.6	-69.01	0.1	74	39	34	-	Abel Monteagudo; Oliver Phillips; Percy Núñez Vargas
CLS-03	-12.61	-69.02	0.1	54	31	29	-	Abel Monteagudo; Oliver Phillips; Percy Núñez Vargas
CLT-01	-12.82	-69.35	0.1	62	44	39	-	Abel Monteagudo; Oliver Phillips; Percy Núñez Vargas
CLT-02	-12.8	-69.34	0.1	68	46	39	-	Abel Monteagudo; Oliver Phillips; Percy Núñez Vargas
CLT-03	-12.84	-69.29	0.1	75	40	36	-	Abel Monteagudo; Oliver Phillips; Percy Núñez Vargas
CLT-04	-12.83	-69.27	0.1	55	42	37	-	Abel Monteagudo; Oliver Phillips; Percy Núñez Vargas
CLT-05	-12.82	-69.35	0.1	76	49	41	-	Abel Monteagudo; Oliver Phillips; Percy Núñez Vargas
CLT-06	-12.88	-69.28	0.1	66	46	39	-	Abel Monteagudo; Oliver Phillips; Percy Núñez Vargas
CLT-07	-12.85	-69.29	0.1	55	38	35	-	Abel Monteagudo; Oliver Phillips; Percy Núñez Vargas

Plot Code	Lat.	Long.	Plot size ha	Nº Ind.	Nº Species	Nº Genera	Multiple census	Data contributors
CLT-08	-12.83	-69.26	0.1	56	42	39	-	Abel Monteagudo; Oliver Phillips; Percy Núñez Vargas
CLT-09	-12.82	-69.3	0.1	68	39	37	-	Abel Monteagudo; Oliver Phillips; Percy Núñez Vargas
CLV-01	-12.4	-68.82	0.1	75	51	39	-	Abel Monteagudo; Oliver Phillips
CLV-02	-12.39	-68.79	0.1	67	51	44	-	Abel Monteagudo; Oliver Phillips
CLV-03	-12.41	-68.86	0.1	77	42	37	-	Abel Monteagudo; Oliver Phillips
CLV-04	-12.36	-68.8	0.1	80	43	36	-	Abel Monteagudo; Oliver Phillips
CLV-05	-12.43	-68.8	0.1	64	37	32	-	Abel Monteagudo; Oliver Phillips
CLV-06	-12.46	-68.81	0.1	60	30	30	-	Abel Monteagudo; Oliver Phillips
CLV-07	-12.47	-68.8	0.1	57	37	35	-	Abel Monteagudo; Oliver Phillips
CLV-08	-12.45	-68.81	0.1	63	37	33	-	Abel Monteagudo; Oliver Phillips
CNG-01	0.83	-66.17	1	535	91	62	-	Alwyn Gentry
CNS-01	-12.56	-68.71	0.1	70	33	30	-	Abel Monteagudo; Oliver Phillips; Percy Núñez Vargas
CNS-02	-12.56	-68.7	0.1	71	42	35	-	Abel Monteagudo; Oliver Phillips; Percy Núñez Vargas
CNS-03	-12.56	-68.72	0.1	59	36	32	-	Abel Monteagudo; Oliver Phillips; Percy Núñez Vargas
CNS-04	-12.59	-68.73	0.1	80	33	25	-	Abel Monteagudo; Oliver Phillips; Percy Núñez Vargas
CNS-05	-12.6	-68.72	0.1	61	36	35	-	Abel Monteagudo; Oliver Phillips; Percy Núñez Vargas
CNS-06	-12.61	-68.73	0.1	58	44	37	-	Abel Monteagudo; Oliver Phillips; Percy Núñez Vargas
CNS-07	-12.6	-68.75	0.1	59	37	34	-	Abel Monteagudo; Oliver Phillips; Percy Núñez Vargas
CNS-08	-12.59	-68.71	0.1	67	37	37	-	Abel Monteagudo; Oliver Phillips; Percy Núñez Vargas
CNS-09	-12.63	-68.75	0.1	90	41	34	-	Abel Monteagudo; Oliver Phillips; Percy Núñez Vargas
CNS-10	-12.65	-68.74	0.1	57	34	29	-	Abel Monteagudo; Oliver Phillips; Percy Núñez Vargas
CNT-01	-12.5	-69.42	0.1	54	38	29	-	Abel Monteagudo; Fernando Cornejo; Oliver Phillips; Percy Núñez Vargas
CNT-02	-12.49	-69.41	0.1	60	39	35	-	Abel Monteagudo; Fernando Cornejo; Oliver Phillips; Percy Núñez Vargas
CNT-03	-12.49	-69.41	0.1	61	12	11	-	Abel Monteagudo; Fernando Cornejo; Oliver Phillips; Percy Núñez Vargas
CNT-04	-12.54	-69.39	0.1	44	27	26	-	Abel Monteagudo; Fernando Cornejo; Oliver Phillips; Percy Núñez Vargas
CNT-05	-12.5	-69.37	0.1	63	38	32	-	Abel Monteagudo; Fernando Cornejo; Oliver Phillips; Percy Núñez Vargas
CNT-06	-12.48	-69.39	0.1	68	41	38	-	Abel Monteagudo; Fernando Cornejo; Oliver Phillips; Percy Núñez Vargas
CNT-07	-12.53	-69.48	0.1	53	39	30	-	Abel Monteagudo; Fernando Cornejo; Oliver Phillips; Percy Núñez Vargas
CNT-08	-12.53	-69.48	0.1	58	26	26	-	Abel Monteagudo; Fernando Cornejo; Oliver Phillips; Percy Núñez Vargas
CNT-09	-12.53	-69.48	0.1	58	38	30	-	Abel Monteagudo; Fernando Cornejo; Oliver Phillips; Percy Núñez Vargas
CNT-10	-12.53	-69.48	0.1	55	36	33	-	Abel Monteagudo; Fernando Cornejo; Oliver Phillips; Percy Núñez Vargas
COL-01	2.1	-67.1	0.1	126	34	29	-	Gerardo Aymard
CON-01	-4.12	-72.92	0.1	57	43	37	-	Oliver Phillips; Rodolfo Vasquez

Plot Code	Lat.	Long.	Plot size ha	Nº Ind.	Nº Species	Nº Genera	Multiple census	Data contributors
CON-02	-4.12	-72.93	0.1	60	45	37	-	Oliver Phillips; Rodolfo Vasquez
CON-03	-4.12	-72.92	0.1	59	40	36	-	Oliver Phillips; Rodolfo Vasquez
CON-11	-4.25	-72.75	0.1	79	52	48	-	Alwyn Gentry
CON-12	-4.16	-72.96	0.1	79	55	46	-	Oliver Phillips; Rodolfo Vasquez
CON-13	-4.15	-72.96	0.1	63	36	30	-	Oliver Phillips; Rodolfo Vasquez
CPA-01	-12.47	-69.21	0.1	55	33	29	-	Abel Monteagudo; Oliver Phillips; Percy Núñez Vargas
CPA-02	-12.48	-69.2	0.1	62	40	34	-	Abel Monteagudo; Oliver Phillips; Percy Núñez Vargas
CPA-03	-12.47	-69.23	0.1	58	34	29	-	Abel Monteagudo; Oliver Phillips; Percy Núñez Vargas
CPA-04	-12.48	-69.21	0.1	73	41	36	-	Abel Monteagudo; Oliver Phillips; Percy Núñez Vargas
CPA-05	-12.45	-69.2	0.1	69	50	42	-	Abel Monteagudo; Oliver Phillips; Percy Núñez Vargas
CPA-06	-12.46	-69.2	0.1	49	37	34	-	Abel Monteagudo; Oliver Phillips; Percy Núñez Vargas
CPA-07	-12.48	-69.22	0.1	66	43	36	-	Abel Monteagudo; Oliver Phillips; Percy Núñez Vargas
CPA-08	-12.5	-69.22	0.1	49	29	26	-	Abel Monteagudo; Oliver Phillips; Percy Núñez Vargas
CPA-09	-12.48	-69.2	0.1	63	40	39	-	Abel Monteagudo; Oliver Phillips; Percy Núñez Vargas
CPP-01	-1.84	-47.1	1	497	132	78	X	Ima Vieira
CPP-02	-1.84	-47.1	1	465	125	80	-	Ima Vieira
CPR-01	-12.51	-68.75	0.1	66	30	22	-	Abel Monteagudo; Oliver Phillips; Percy Núñez Vargas
CPR-02	-12.52	-68.73	0.1	66	40	35	-	Abel Monteagudo; Oliver Phillips; Percy Núñez Vargas
CPR-03	-12.5	-68.78	0.1	75	38	30	-	Abel Monteagudo; Oliver Phillips; Percy Núñez Vargas
CPR-04	-12.5	-68.75	0.1	61	30	28	-	Abel Monteagudo; Oliver Phillips; Percy Núñez Vargas
CPR-05	-12.55	-68.77	0.1	172	25	22	-	Abel Monteagudo; Oliver Phillips; Percy Núñez Vargas
CPR-06	-12.49	-68.76	0.1	64	27	27	-	Abel Monteagudo; Oliver Phillips; Percy Núñez Vargas
CPR-07	-12.5	-68.78	0.1	71	38	36	-	Abel Monteagudo; Oliver Phillips; Percy Núñez Vargas
CPR-08	-12.52	-68.75	0.1	84	35	30	-	Abel Monteagudo; Oliver Phillips; Percy Núñez Vargas
CPR-09	-12.53	-68.76	0.1	65	29	26	-	Abel Monteagudo; Oliver Phillips; Percy Núñez Vargas
CPR-10	-12.53	-68.72	0.1	126	36	30	-	Abel Monteagudo; Oliver Phillips; Percy Núñez Vargas
CPR-11	-12.48	-68.76	0.1	73	22	22	-	Abel Monteagudo; Oliver Phillips; Percy Núñez Vargas
CPZ-01	2.03	-67.07	0.1	77	31	24	-	Gerardo Aymard
CRP-01	-14.54	-61.5	1	768	69	48	X	Abel Monteagudo; Roel Brienen; Alejandro Murakami-Araujo;Luzmila Arroyo; Timothy Killeen
CRP-02	-14.54	-61.5	1	717	80	58	X	Abel Monteagudo; Roel Brienen; Alejandro Murakami-Araujo;Luzmila Arroyo; Timothy Killeen
CRZ-01	8.83	-71.86	1	563	21	19	X	Jean-Pierre Veillon; Oliver Phillips

Plot Code	Lat.	Long.	Plot size ha	Nº Ind.	Nº Species	Nº Genera	Multiple census	Data contributors
CSM-01	2.58	-67.12	1	496	61	48	-	Gerardo Aymard
CUZ-01	-12.54	-69.06	1	912	185	111	X	Abel Monteagudo; Oliver Phillips; Rodolfo Vasquez
CUZ-02	-12.54	-69.06	1	805	164	108	X	Abel Monteagudo; Oliver Phillips; Rodolfo Vasquez
CUZ-03	-12.53	-69.05	1	752	168	109	X	Abel Monteagudo; Oliver Phillips; Rodolfo Vasquez
CUZ-04	-12.54	-69.05	1	971	211	115	X	Abel Monteagudo; Oliver Phillips; Rodolfo Vasquez
CUZ-10	-12.58	-69.15	0.1	84	41	37	-	Alwyn Gentry; Percy Núñez Vargas
DOI-01	-10.57	-68.32	1	669	208	138	X	Ted Feldpausch; Marcos Silveira; Tim Baker; Juliana Stropp; Wenderson Castro
DOI-02	-10.55	-68.31	1	514	145	102	X	Ted Feldpausch; Marcos Silveira; Tim Baker; Juliana Stropp; Wenderson Castro
DUR-01	0.25	-76.75	0.04	37	32	28	-	Alwyn Gentry
EBB-01	-14.78	-66.34	1	611	45	41	X	Gerardo Aymard; James Comiskey
EBB-02	-14.77	-66.35	1	618	57	50	X	Gerardo Aymard; James Comiskey
EBB-03	-14.84	-66.34	1	522	53	47	X	Gerardo Aymard; James Comiskey
EBB-05	-14.76	-66.34	1	467	34	31	X	Gerardo Aymard; James Comiskey
EBB-07	-14.86	-66.32	1	588	57	54	X	Gerardo Aymard; James Comiskey
EBB-08	-14.85	-66.34	1	398	54	49	X	Gerardo Aymard; James Comiskey
EBB-09	-14.73	-66.32	1	562	36	32	X	Gerardo Aymard; James Comiskey
EBB-10	-14.89	-66.59	1	674	43	40	X	Gerardo Aymard; James Comiskey
EBB-11	-14.85	-66.36	1	602	16	13	X	Gerardo Aymard; James Comiskey
EBB-12	-14.64	-66.06	1	504	32	31	-	Gerardo Aymard; James Comiskey
EBB-13	-14.74	-66.27	1	448	38	33	X	Gerardo Aymard; James Comiskey
EBB-14	-14.74	-66.56	1	488	46	41	X	Gerardo Aymard; James Comiskey
ELD-01	6.11	-61.41	0.25	163	46	37	X	Emilio Vilanova; Hirma Ramirez-Angulo; Armando Torres-Lezama; Geertje van der Heijden; Oliver Phillips
ELD-02	6.11	-61.41	0.25	156	36	30	X	Emilio Vilanova; Hirma Ramirez-Angulo; Armando Torres-Lezama; Geertje van der Heijden; Oliver Phillips
ELD-03	6.09	-61.4	0.25	248	64	49	X	Emilio Vilanova; Hirma Ramirez-Angulo; Armando Torres-Lezama; Geertje van der Heijden; Oliver Phillips
ELD-04	6.09	-61.35	0.25	252	59	45	X	Emilio Vilanova; Hirma Ramirez-Angulo; Armando Torres-Lezama; Geertje van der Heijden; Oliver Phillips
EMC-01	8.01	-70.55	1	278	24	21	X	Emilio Vilanova
EMC-02	8.01	-70.55	1	245	23	22	X	Emilio Vilanova
EMC-03	8.01	-70.55	1	452	23	20	X	Emilio Vilanova
ENT-01	-14.63	-60.7	0.1	65	37	36	-	Alwyn Gentry

Plot Code	Lat.	Long.	Plot size ha	Nº Ind.	Nº Species	Nº Genera	Multiple census	Data contributors
FEC-01	-10.07	-67.62	1	657	163	116	X	Foster Brown; Marcos Silveira; Oliver Phillips, Plínio Barbosa Camargo; Simone Aparecida Vieira, Ted Feldpausch, Wendeson Castro
FLO-01	-12.81	-51.85	1	684	85	56	X	Beatriz Marimon, Ben Hur Marimon Junior; Edmar de Oliveira; Leandro Maracahipes; Ted Feldpausch
FLO-02	-12.76	-51.88	1	562	63	44	X	Beatriz Marimon, Ben Hur Marimon Junior; Edmar de Oliveira; Leandro Maracahipes; Ted Feldpausch
FMH-01	5.17	-58.69	1	494	58	41	X	Ted Feldpausch; Hans ter Steege; Eric Arets
FMH-02	5.17	-58.69	1	421	47	35	X	Hans ter Steege; James Singh; Roderick Zagt; Oliver Phillips; Roel Brienen; Ted Feldpausch
FMH-03	5.18	-58.7	1	661	57	39	X	Hans ter Steege; James Singh; Roderick Zagt; Oliver Phillips; Roel Brienen; Ted Feldpausch
FOB-01	-13.57	-61.02	1	387	25	23	-	Luzmila Arroyo; Timothy Killeen
GAB-01	1.7	-66.98	0.1	70	36	30	-	Gerardo Aymard
GAL-01	5.8	-67.33	0.1	115	50	43	-	Gerardo Aymard
GMT-01	-1.11	-47.8	1	544	149	96	X	Tim Baker
HEA-01	-12.83	-68.83	0.1	65	40	35	-	Alwyn Gentry
HSP-01	8.5	-69	1	417	26	25	X	Jean-Pierre Veillon
HUM-01	-8.83	-75	0.1	75	36	31	-	Alwyn Gentry; Kenneth Young
IMA-01	7.44	-61.17	1	526	68	56	-	Gerardo Aymard; Oliver Phillips
IMA-02	7.45	-61.17	1	490	59	51	-	Gerardo Aymard; Oliver Phillips
IND-01	-3.52	-72.85	1	591	171	107	X	John Pipoly; Rodolfo Vasquez
IND-10	-3.51	-73.06	0.1	89	56	50	-	Alwyn Gentry
INF-01	-12.73	-69.7	1.3	912	173	113	X	Miguel Alexiades; Oliver Phillips
IPR-01	13.35	-81.36	0.1	91	22	22	-	Alwyn Gentry
ISR-01	9.92	-75.81	1	628	19	18	X	Esteban Álvarez Dávila
IVC-01	1.92	-67.03	0.1	84	50	41	-	Gerardo Aymard
IVC-02	1.92	-67.03	0.1	100	22	19	-	Gerardo Aymard
IVC-03	1.92	-67.03	0.1	81	30	22	-	Gerardo Aymard
IWO-03	4.53	-58.78	1	580	72	49	X	Anand Roopsind; Oliver Phillips; Raquel Thomas
IWO-09	4.61	-58.73	1	695	53	37	X	Anand Roopsind; Oliver Phillips; Raquel Thomas
IWO-11	4.62	-58.72	1	454	64	45	X	Anand Roopsind; Oliver Phillips; Raquel Thomas
IWO-12	4.73	-58.72	1	471	83	55	X	Anand Roopsind; Oliver Phillips; Raquel Thomas
IWO-21	4.63	-58.74	1	626	43	32	X	Anand Roopsind; Oliver Phillips; Raquel Thomas; Roel Brienen; Ted Feldpausch

Plot Code	Lat.	Long.	Plot size ha	Nº Ind.	Nº Species	Nº Genera	Multiple census	Data contributors
IWO-22	4.62	-58.72	1	471	70	49	X	Anand Roopsind; Oliver Phillips; Raquel Thomas; Roel Brienen; Ted Feldpausch
JAC-01	-2.61	-60.21	5	3565	194	134	X	Niro Higuchi
JAC-02	-2.62	-60.2	5	3440	205	137	X	Niro Higuchi
JAM-01	-4.67	-66.17	4	3118	431	202	-	Antonio S. Lima
JAS-02	-1.07	-77.62	1	1162	274	147	X	Roel Brienen; David Neill
JAS-03	-1.08	-77.61	1	909	267	143	X	Roel Brienen; David Neill
JAS-04	-1.07	-77.61	1	996	231	137	X	Roel Brienen; David Neill
JAS-05	-1.06	-77.62	1	703	193	121	X	David Neill
JAS-10	-1.06	-77.6	0.1	76	59	52	-	Alwyn Gentry; David Neill
JAU-01	-1.1	-79.63	0.1	64	30	29	-	Alwyn Gentry; David Neill
JBU-01	-1.14	-47.7	1	528	144	98	X	Ima Vieira
JEN-01	-4.91	-73.75	0.1	71	52	46	-	Rodolfo Vasquez
JEN-11	-4.88	-73.63	1	680	254	122	X	Euridice Honorio
JEN-12	-4.9	-73.63	1	798	134	84	X	Euridice Honorio
JEN-13	-4.92	-73.54	1	680	192	116	X	Eurídice Honorio
JEN-14	-4.84	-73.83	0.5	202	32	28	-	Eurídice Honorio
JEN-15	-4.84	-73.65	0.5	283	69	57	-	Eurídice Honorio
JFR-01	-10.48	-58.47	0.93	472	39	36	-	Ted Feldpausch
JFR-02	-10.55	-58.49	0.525	241	31	30	-	Ted Feldpausch
JFR-03	-10.48	-58.52	1.025	573	45	43	-	Ted Feldpausch
JFR-04	-10.48	-58.48	1	569	40	39	-	Ted Feldpausch
JFR-05	-10.48	-58.48	1	555	38	35	-	Ted Feldpausch
JFR-06	-10.47	-58.49	1	507	37	35	-	Ted Feldpausch
JFR-07	-10.48	-58.5	1.025	476	36	36	-	Ted Feldpausch
JFR-08	-10.47	-58.5	1	505	34	33	-	Ted Feldpausch
JFR-09	-10.47	-58.51	0.975	604	32	31	-	Ted Feldpausch
JRI-01	-0.89	-52.19	1	666	133	99	X	Natalino Silva
JUY-01	-2.13	-76.2	1	620	208	126	-	Kenneth Young; Ophelia Wang
LAS-01	-12.56	-70.11	2	1112	137	108	X	Fernando Valverde; Nigel Pitman
LAS-02	-12.57	-70.09	1	650	179	119	X	Fernando Valverde; Nigel Pitman
LAS-03	-12.53	-70.08	2	1334	280	135	X	Fernando Valverde; Nigel Pitman
LCL-01	9.96	-75.16	0.1	54	29	26	-	Alwyn Gentry
LFB-01	-14.58	-60.83	1	899	106	80	X	Roel Brienen

Plot Code	Lat.	Long.	Plot size ha	Nº Ind.	Nº Species	Nº Genera	Multiple census	Data contributors
LFB-02	-14.58	-60.83	1	828	91	67	X	Roel Brienen
LJV-01	2.07	-67.08	0.1	88	38	35	-	Gerardo Aymard
LMS-01	4.27	-54.75	1	638	117	88	-	Hans ter Steege
LMS-02	4.26	-54.74	1	494	105	90	-	Hans ter Steege
LMS-03	4.27	-54.74	1	601	117	92	-	Hans ter Steege
LMS-04	4.25	-54.73	1	524	107	89	-	Hans ter Steege
LMS-05	4.25	-54.73	1	981	24	23	-	Hans ter Steege
LMS-06	4.26	-54.78	1	477	86	69	-	Hans ter Steege
LMS-07	4.27	-54.78	1	476	85	71	-	Hans ter Steege
LMS-08	4.27	-54.75	1	489	90	70	-	Hans ter Steege
LOR-01	-3.06	-69.99	1	874	275	141	X	Adriana Prieto; Agustin Rudas
LOR-02	-3.06	-69.99	0.52	401	168	100	X	Adriana Prieto; Agustin Rudas
LOR-03	-3.06	-69.99	0.48	413	178	107	X	Adriana Prieto; Agustin Rudas
LSL-01	-14.4	-61.14	1	779	100	68	X	Alejandro Araujo-Murakami; Jon Lloyd; Luzmila Arroyo; Timothy Killeen; Roel Brienen
LSL-02	-14.4	-61.14	1	810	97	72	X	Alejandro Araujo-Murakami; Jon Lloyd; Luzmila Arroyo; Timothy Killeen; Roel Brienen
LST-01	4.91	-74.83	0.1	96	20	19	-	Alwyn Gentry
MAJ-01	1.9	-67.03	0.1	65	24	20	-	Gerardo Aymard
MAJ-02	1.9	-67.05	0.1	105	45	39	-	Gerardo Aymard
MAS-01	7.55	-76.08	0.1	77	45	41	-	Alwyn Gentry
MBT-01	-10.07	-65.89	1	548	160	101	X	Marisol Toledo; Roel Brienen; Guido Pardo; Juan Licona
MBT-02	-10.05	-65.89	1	505	152	93	X	Marisol Toledo; Roel Brienen; Guido Pardo; Juan Licona
MBT-04	-10.31	-65.55	1	560	155	101	X	Marisol Toledo; Roel Brienen; Guido Pardo; Juan Licona
MBT-05	-10.03	-65.63	1	567	204	114	X	Marisol Toledo; Roel Brienen; Guido Pardo; Juan Licona
MBT-06	-10.04	-65.64	1	541	180	115	X	Marisol Toledo; Roel Brienen; Guido Pardo; Juan Licona
MBT-07	-9.91	-65.74	1	548	157	94	X	Marisol Toledo; Roel Brienen; Guido Pardo; Juan Licona
MBT-08	-9.94	-65.75	1	513	157	99	X	Marisol Toledo; Roel Brienen; Guido Pardo; Juan Licona
MCB-01	-1.44	-48.41	2	856	137	94	-	Rafael Salomão
MIN-01	-8.57	-72.9	1	921	205	136	X	Ted Feldpausch; Marcos Silveira; Jorcely Barroso
MNU-01	-11.89	-71.41	2.25	2100	261	158	X	John Terborgh; Roel Brienen; Fernando Valverde
MNU-03	-11.9	-71.4	2	2168	308	179	X	John Terborgh; Roel Brienen; Fernando Valverde
MNU-04	-11.9	-71.4	2	1792	339	191	X	John Terborgh; Roel Brienen; Fernando Valverde
MNU-05	-11.88	-71.41	2	1739	219	132	X	John Terborgh; Roel Brienen; Fernando Valverde
MNU-06	-11.89	-71.4	2.25	1707	216	131	X	John Terborgh; Roel Brienen; Fernando Valverde

Plot Code	Lat.	Long.	Plot size ha	Nº Ind.	Nº Species	Nº Genera	Multiple census	Data contributors
MNU-08	-12	-71.24	2	1517	199	120	X	John Terborgh; Roel Brienen; Fernando Valverde
MNU-09	-12.04	-71.21	2	1800	183	107	X	John Terborgh; Roel Brienen; Fernando Valverde
MNU-10	-11.85	-71.31	0.1	73	55	44	-	John Terborgh; Roel Brienen; Fernando Valverde
MRB-01	-5.73	-49.05	2	1199	153	108	X	Rafael Salomão
MRB-02	-5.72	-49.03	2	1201	152	107	X	Rafael Salomão
MRB-03	-5.7	-49	2	1147	138	104	X	Rafael Salomão
MSH-01	-3.78	-73.5	1	894	253	141	X	Alwyn Gentry; Oliver Phillips; Rodolfo Vasquez
MSH-10	-3.78	-73.5	0.1	83	60	46	-	Alwyn Gentry
MSH-11	-3.78	-73.5	0.1	67	45	38	-	Alwyn Gentry
MSH-12	-3.78	-73.5	0.1	67	39	32	-	Alwyn Gentry
MTH-01	-8.88	-72.79	1	734	171	123	X	Ted Feldpausch; Marcos Silveira; Jose Barroso; Wenderson Castro
MVE-01	-15.01	-61.13	1	567	79	66	-	Luzmila Arroyo; Timothy Killeen
NCR-01	-14.64	-61.16	1	566	58	39	-	Luzmila Arroyo; Timothy Killeen
NCR-02	-14.71	-61.15	1	590	65	52	-	Luzmila Arroyo; Timothy Killeen
NEB-01	0.83	-66.18	0.1	62	26	21	-	Gerardo Aymard
NEB-02	0.83	-66.18	0.1	59	24	21	-	Gerardo Aymard
NLT-03	-13.66	-60.82	1	919	76	57	-	Luzmila Arroyo; Timothy Killeen
NMS-01	4.78	-54.62	1	475	90	77	-	Hans ter Steege
NMS-02	4.82	-54.61	0.55	256	72	59	-	Hans ter Steege
NMS-03	4.82	-54.6	1	497	105	80	-	Hans ter Steege
NMS-04	4.93	-54.52	1	739	116	91	-	Hans ter Steege
NMS-05	4.93	-54.52	1	810	117	90	-	Hans ter Steege
NMS-06	4.83	-54.61	1	607	106	87	-	Hans ter Steege
NMU-01	-10.65	-66.76	0.1	80	40	35	-	Alwyn Gentry
NOU-01	4.09	-52.67	1	658	94	68	X	Jerome Chave; Ted Feldpausch; Chris Baraloto
NOU-02	4.09	-52.67	1	612	186	106	X	Jerome Chave; Ted Feldpausch; Chris Baraloto
NOU-03	4.09	-52.68	1	557	130	79	X	Jerome Chave; Ted Feldpausch; Chris Baraloto
NOU-04	4.09	-52.68	1	596	112	80	X	Jerome Chave; Ted Feldpausch; Chris Baraloto
NOU-05	4.09	-52.68	1	680	114	82	X	Jerome Chave; Ted Feldpausch; Chris Baraloto
NOU-06	4.09	-52.68	1	642	106	82	X	Jerome Chave; Ted Feldpausch; Chris Baraloto
NOU-07	4.08	-52.68	1	648	109	79	X	Jerome Chave; Ted Feldpausch; Chris Baraloto
NOU-08	4.08	-52.68	1	654	87	69	X	Jerome Chave; Ted Feldpausch; Chris Baraloto
NOU-09	4.08	-52.68	1	669	64	48	X	Jerome Chave; Ted Feldpausch; Chris Baraloto

Plot Code	Lat.	Long.	Plot size ha	Nº Ind.	Nº Species	Nº Genera	Multiple census	Data contributors
NOU-10	4.09	-52.68	1	672	73	50	X	Jerome Chave; Ted Feldpausch; Chris Baraloto
NOU-11	4.08	-52.68	1	633	202	109	X	Jerome Chave; Ted Feldpausch; Chris Baraloto
NOU-12	4.08	-52.68	1	635	205	109	X	Jerome Chave; Ted Feldpausch; Chris Baraloto
NOU-13	4.08	-52.68	1	643	211	113	X	Jerome Chave; Ted Feldpausch; Chris Baraloto
NOU-14	4.08	-52.68	1	676	201	105	X	Jerome Chave; Ted Feldpausch; Chris Baraloto
NOU-15	4.08	-52.68	1	621	200	103	X	Jerome Chave; Ted Feldpausch; Chris Baraloto
NOU-16	4.08	-52.68	1	696	183	105	X	Jerome Chave; Ted Feldpausch; Chris Baraloto
NOU-17	4.08	-52.68	1	653	194	110	X	Jerome Chave; Ted Feldpausch; Chris Baraloto
NOU-18	4.08	-52.68	1	665	222	111	X	Jerome Chave; Ted Feldpausch; Chris Baraloto
NOU-19	4.08	-52.68	1	655	209	103	X	Jerome Chave; Ted Feldpausch; Chris Baraloto
NOU-20	4.08	-52.68	1	650	188	108	X	Jerome Chave; Ted Feldpausch; Chris Baraloto
NOU-21	4.08	-52.68	1	625	173	93	X	Jerome Chave; Ted Feldpausch; Chris Baraloto
NOU-22	4.08	-52.67	1	600	177	106	X	Jerome Chave; Ted Feldpausch; Chris Baraloto
PAB-01	5.27	-52.92	6.25	4675	320	155	X	Bruno Herault; Chris Baraloto
PAB-02	5.27	-52.92	25	16210	419	187	X	Bruno Herault; Chris Baraloto
PAK-01	-11.94	-71.28	1	919	178	117	X	James Comiskey; Oliver Phillips; Patricia Alvarez Loayza
PAK-02	-11.97	-71.27	1	1163	204	129	X	James Comiskey; Oliver Phillips; Patricia Alvarez Loayza
PAR-20	5.28	-52.92	0.49	296	99	69	X	Damien Bonal
PAR-21	5.28	-52.92	0.49	291	107	78	X	Damien Bonal
PAR-22	5.28	-52.92	0.49	275	94	71	X	Damien Bonal
PAR-23	5.28	-52.92	0.49	264	94	69	X	Damien Bonal
PAR-24	5.28	-52.92	0.49	279	103	75	X	Damien Bonal
PAR-25	5.28	-52.92	0.49	513	145	96	X	Damien Bonal
PAR-26	5.28	-52.92	0.49	324	75	59	X	Damien Bonal
PAR-27	5.28	-52.92	0.49	266	96	67	X	Damien Bonal
PAR-28	5.28	-52.92	0.49	357	113	84	X	Damien Bonal
PAR-29	5.28	-52.92	0.49	348	119	77	X	Damien Bonal
PAY-01	-0.45	-77.03	1	653	200	122	-	Carlos Céron; David Neill; Nigel Pitman; Walter Palacios
PBS-01	-12.65	-68.74	0.1	89	47	40	-	Abel Monteagudo; Oliver Phillips; Percy Núñez Vargas
PBS-02	-12.66	-68.75	0.1	73	36	32	-	Abel Monteagudo; Oliver Phillips; Percy Núñez Vargas
PBS-03	-12.73	-68.78	0.1	61	40	37	-	Abel Monteagudo; Oliver Phillips; Percy Núñez Vargas
PBS-04	-12.72	-68.81	0.1	124	46	38	-	Abel Monteagudo; Oliver Phillips; Percy Núñez Vargas
PEA-02	-12.32	-50.74	1	1008	36	33	X	Beatriz Marimon; Ben Hur Marimon Junior

Plot Code	Lat.	Long.	Plot size ha	Nº Ind.	Nº Species	Nº Genera	Multiple census	Data contributors
PEA-03	-12.38	-50.89	1	1579	21	18	-	Beatriz Marimon; Ben Hur Marimon Junior; Eddie Lenza de Oliveira
PEA-04	-12.42	-50.71	1	1278	29	27	-	Beatriz Marimon; Ben Hur Marimon Junior; Eddie Lenza de Oliveira
PEA-05	-11.9	-50.75	1	966	33	30	-	Beatriz Marimon; Ben Hur Marimon Junior; Eddie Lenza de Oliveira
PEA-06	-11.92	-50.71	1	922	34	32	-	Beatriz Marimon; Ben Hur Marimon Junior; Eddie Lenza de Oliveira
PEA-07	-12.48	-50.9	1	472	11	11	-	Beatriz Marimon; Ben Hur Marimon Junior; Eddie Lenza de Oliveira
PEA-08	-12.54	-50.74	1	426	11	10	-	Beatriz Marimon; Ben Hur Marimon Junior; Eddie Lenza de Oliveira
PGP-01	2	-66.63	0.1	90	25	24	-	Gerardo Aymard
PIB-05	5.02	-58.62	1	513	82	54	X	Hans ter Steege; James Singh; Peter van de Hout
PIB-06	5.01	-58.62	1	576	89	56	X	Hans ter Steege; James Singh; Peter van de Hout
PIB-12	5.03	-58.6	1	451	103	61	X	Hans ter Steege; James Singh; Peter van de Hout
PNY-04	-10.34	-75.25	1	628	183	114	X	Abel Monteagudo; Oliver Phillips; Rodolfo Vasquez;
PNY-05	-10.35	-75.25	1	616	168	90	X	Abel Monteagudo; Oliver Phillips; Rodolfo Vasquez;
PNY-06	-10.36	-75.25	1	503	135	78	X	Abel Monteagudo; Oliver Phillips; Rodolfo Vasquez;
PNY-07	-10.35	-75.26	1	566	199	117	X	Abel Monteagudo; Oliver Phillips; Rodolfo Vasquez;
POR-01	-10.82	-68.77	1	767	189	131	X	Marcos Silveira; Oliver Phillips; Ted Feldpausch; Tim Baker
POR-02	-10.8	-68.77	1	762	188	122	X	Marcos Silveira; Oliver Phillips; Ted Feldpausch; Tim Baker
PPB-01	-1.18	-47.32	1	499	148	101	X	Rafael Salomão
PPB-02	-1.18	-47.32	1	583	180	114	X	Rafael Salomão
PPB-03	-1.18	-47.32	1	549	147	97	X	Rafael Salomão
PTA-01	5.11	-67.74	1	500	97	68	-	Gerardo Aymard; Oliver Phillips
PTA-02	5.84	-67.45	1	436	84	55	-	Gerardo Aymard; Oliver Phillips
PTA-03	5.11	-67.74	1	593	105	67	-	Gerardo Aymard; Oliver Phillips
PTA-05	5.84	-67.45	1	383	69	57	-	Gerardo Aymard; Oliver Phillips
PTA-06	5.84	-67.45	1	365	52	46	-	Gerardo Aymard; Oliver Phillips
PTA-07	5.78	-67.46	1	504	72	58	-	Gerardo Aymard; Oliver Phillips
PTA-08	5.78	-67.46	1	478	67	52	-	Gerardo Aymard; Oliver Phillips
PTA-12	5.08	-67.67	0.1	125	45	39	-	Gerardo Aymard; Oliver Phillips
PTB-01	-1.17	-56.41	1	509	199	110	X	Rafael Salomão
PTB-02	-1.48	-56.39	1	588	203	97	X	Rafael Salomão
PTN-01	6.12	-74.67	1	673	127	98	X	Esteban Alvarez
QUI-01	-3.83	-73.32	0.5	411	16	14	-	Eurídice Honorio; Tim Baker
RAY-01	8.33	-74.91	0.1	84	52	46	-	Alwyn Gentry
RBR-01	-11	-61.95	1	560	157	114	-	Rafael Salomão

Plot Code	Lat.	Long.	Plot size ha	Nº Ind.	Nº Species	Nº Genera	Multiple census	Data contributors
RCS-05	-9.62	-74.93	1	595	180	112	-	Abel Monteagudo; Oliver Phillips
REQ-01	-4.91	-73.82	0.5	260	51	40	-	Eurídice Honorio
REQ-04	-4.88	-73.79	0.5	237	37	34	-	Eurídice Honorio
REQ-05	-4.81	-73.82	0.5	205	45	41	-	Eurídice Honorio
REQ-13	-4.87	-73.65	0.5	307	57	49	-	Eurídice Honorio
RET-05	-10.97	-65.72	1	816	130	83	X	Marisol Toledo;Roel Brienen; Eric Arets; Laurens Poorter; Marielos Pena Claros; Vincent Vos; Rene Boot; Guido Pardo
RET-06	-10.97	-65.72	1	742	129	85	X	Marisol Toledo;Roel Brienen; Eric Arets; Laurens Poorter; Marielos Pena Claros; Vincent Vos; Rene Boot; Guido Pardo
RET-08	-10.97	-65.72	1	713	111	79	X	Marisol Toledo;Roel Brienen; Eric Arets; Laurens Poorter; Marielos Pena Claros; Vincent Vos; Rene Boot; Guido Pardo
RET-09	-10.97	-65.72	1	628	112	76	X	Marisol Toledo;Roel Brienen; Eric Arets; Laurens Poorter; Marielos Pena Claros; Vincent Vos; Rene Boot; Guido Pardo
RFE-34	-4.01	-73.45	0.04	23	19	17	-	Kalle Ruokolainen
RFE-35	-4.01	-73.45	0.04	33	26	22	-	Kalle Ruokolainen
RFE-36	-4.01	-73.45	0.04	27	25	24	-	Kalle Ruokolainen
RFE-37	-4.01	-73.45	0.04	27	25	24	-	Kalle Ruokolainen
RFH-01	-9.75	-67.67	1	472	148	111	X	Foster Brown; Marcos Silveira; Ted Feldpausch; Wenderson Castro
RGE-15	-3.61	-73.3	0.04	21	18	18	-	Kalle Ruokolainen
RGE-16	-3.61	-73.3	0.04	25	19	18	-	Kalle Ruokolainen
RGE-17	-3.61	-73.3	0.04	17	12	12	-	Kalle Ruokolainen
RHA-26	-3.51	-72.04	0.04	20	15	11	-	Kalle Ruokolainen
RHA-27	-3.51	-72.04	0.04	19	17	16	-	Kalle Ruokolainen
RHA-28	-3.51	-72.04	0.04	20	16	15	-	Kalle Ruokolainen
RHA-29	-3.51	-72.04	0.04	35	27	25	-	Kalle Ruokolainen
RIO-01	8.11	-61.69	0.25	193	58	47	X	Emilio Vilanova; Hirma Ramirez-Angulo; Armando Torres-Lezama ; Geertje van der Heijden; Oliver Phillips
RIO-02	8.11	-61.69	0.25	219	68	51	X	Emilio Vilanova; Hirma Ramirez-Angulo; Armando Torres-Lezama ; Geertje van der Heijden; Oliver Phillips
RMA-22	-3.6	-72.9	0.04	23	17	15	-	Kalle Ruokolainen
RMA-23	-3.6	-72.9	0.04	22	18	17	-	Kalle Ruokolainen
RMA-24	-3.6	-72.9	0.04	26	19	18	-	Kalle Ruokolainen
RMA-25	-3.6	-72.9	0.04	29	23	21	-	Kalle Ruokolainen

Plot Code	Lat.	Long.	Plot size ha	Nº Ind.	Nº Species	Nº Genera	Multiple census	Data contributors
RMI-05	-3.89	-73.48	0.0625	37	26	21	-	Kalle Ruokolainen
RMI-06	-3.89	-73.48	0.0625	54	35	26	-	Kalle Ruokolainen
RMI-07	-3.89	-73.48	0.0625	31	27	22	-	Kalle Ruokolainen
RMI-08	-3.89	-73.48	0.0625	42	30	29	-	Kalle Ruokolainen
RMI-09	-3.9	-73.48	0.0625	27	18	17	-	Kalle Ruokolainen
RMI-10	-3.9	-73.48	0.0625	32	22	22	-	Kalle Ruokolainen
RMI-11	-3.9	-73.48	0.0625	29	19	15	-	Kalle Ruokolainen
RMI-12	-3.9	-73.48	0.0625	29	21	20	-	Kalle Ruokolainen
RMI-13	-3.9	-73.48	0.0625	40	24	23	-	Kalle Ruokolainen
RMO-01	-3.67	-73.29	0.06251	30	28	24	-	Kalle Ruokolainen
RMO-02	-3.67	-73.29	0.0625	34	22	19	-	Kalle Ruokolainen
RMO-03	-3.66	-73.29	0.0625	27	25	23	-	Kalle Ruokolainen
RMO-04	-3.66	-73.29	0.0625	35	31	27	-	Kalle Ruokolainen
RNA-18	-4.45	-73.59	0.04	20	15	13	-	Kalle Ruokolainen
RNA-19	-4.45	-73.59	0.04	24	21	18	-	Kalle Ruokolainen
RNA-20	-4.45	-73.59	0.04	25	19	16	-	Kalle Ruokolainen
RNA-21	-4.45	-73.59	0.04	23	16	13	-	Kalle Ruokolainen
RNE-01	-9.83	-65.66	0.1	51	33	31	-	Alwyn Gentry
RPA-01	-12.39	-69.36	1	527	155	107	-	John Terborgh; Percy Núñez Vargas
RPE-38	-4.07	-73.46	0.04	22	19	19	-	Kalle Ruokolainen
RPE-39	-4.07	-73.46	0.04	31	26	23	-	Kalle Ruokolainen
RPE-40	-4.07	-73.46	0.04	30	26	24	-	Kalle Ruokolainen
RPE-41	-4.07	-73.46	0.04	18	16	14	-	Kalle Ruokolainen
RPI-01	-12.36	-69.23	1	552	132	96	-	John Terborgh; Percy Núñez Vargas
RPL-01	-0.56	-79.33	0.1	50	23	21	-	Alwyn Gentry
RPL-02	-0.56	-79.33	0.1	55	29	26	-	Alwyn Gentry
RPN-30	-3.88	-73.08	0.04	26	18	16	-	Kalle Ruokolainen
RPN-31	-3.88	-73.08	0.04	29	20	18	-	Kalle Ruokolainen
RPN-32	-3.88	-73.08	0.04	31	24	21	-	Kalle Ruokolainen
RSA-42	-4.09	-73.12	0.04	32	30	21	-	Kalle Ruokolainen
RSA-43	-4.09	-73.12	0.04	31	26	25	-	Kalle Ruokolainen
RSA-44	-4.09	-73.12	0.04	28	23	22	-	Kalle Ruokolainen
RSA-45	-4.09	-73.12	0.04	29	26	24	-	Kalle Ruokolainen

Plot Code	Lat.	Long.	Plot size ha	Nº Ind.	Nº Species	Nº Genera	Multiple census	Data contributors
RSN-46	-4.54	-73.63	0.04	21	17	17	-	Kalle Ruokolainen
RSN-47	-4.54	-73.63	0.04	16	15	15	-	Kalle Ruokolainen
RSN-48	-4.54	-73.63	0.04	24	23	20	-	Kalle Ruokolainen
RSN-49	-4.54	-73.63	0.04	29	22	17	-	Kalle Ruokolainen
RST-01	-9.04	-72.27	1	696	181	125	X	Ted Feldpausch; Marcos Silveira; Jose Barroso
RTA-01	-13.35	-69.66	0.1	72	44	40	-	Alwyn Gentry; Percy Núñez Vargas
RTH-01	-11.37	-69.66	1	525	150	102	-	John Terborgh; Percy Núñez Vargas
RTP-50	-3.78	-73.45	0.04	33	22	22	-	Kalle Ruokolainen
RTP-51	-3.78	-73.45	0.04	27	22	20	-	Kalle Ruokolainen
RTP-52	-3.78	-73.45	0.04	31	27	25	-	Kalle Ruokolainen
RTP-53	-3.78	-73.45	0.04	28	22	21	-	Kalle Ruokolainen
SAA-01	-9.79	-50.43	1	532	90	68	X	Beatriz Marimon; Ben Hur Marimon Junior; Eder Carvalho das Neves; Fernando Elias
SAA-02	-9.64	-50.45	1	546	74	58	-	Beatriz Marimon; Ben Hur Marimon Junior; Eder Carvalho das Neves; Fernando Elias
SAT-01	-9.84	-50.46	1	518	78	63	-	Beatriz Marimon; Ben Hur Marimon Junior; Eder Carvalho das Neves; Fernando Elias
SAW-01	-2.64	-77.15	1	555	118	87	-	Kenneth Young; Ophelia Wang
SCR-04	1.93	-67.04	1	1035	48	34	X	Gerardo Aymard; Jon Lloyd; Oliver Phillips; Rafael Herrera Fernández
SCR-05	1.93	-67.04	1	934	131	80	X	Carlos Quesada; Gerardo Aymard; Oliver Phillips
SCT-01	-17.09	-64.77	1	744	78	58	X	Luzmilla Arroyo; Casimiro Mendoza; Oliver Phillips; Roel Brienen
SCT-06	-17.09	-64.77	1	844	70	55	X	Luzmilla Arroyo; Casimiro Mendoza; Oliver Phillips; Roel Brienen
SHI-01	-1.02	-76.98	1	628	193	120	-	Carlos Céron; Nigel Pitman
SHR-01	-10.31	-75.11	0.1	63	48	40	-	Alwyn Gentry
SIP-01	-11.41	-55.32	1	474	40	39	-	Marcos Silveira
SJO-01	-4.06	-73.2	0.5	580	10	10	-	Eurídice Honorio
SRT-01	-1.46	-47.92	1	531	136	88	X	Ima Vieira
SUC-01	-3.25	-72.91	1	830	378	167	X	Oliver Phillips; Tim Baker; Roel Brienen; Rodolfo Vasquez; Abel Monteagudo
SUC-02	-3.25	-72.9	1	835	355	161	X	Oliver Phillips; Tim Baker; Roel Brienen; Rodolfo Vasquez; Abel Monteagudo
SUC-03	-3.25	-72.92	1	687	94	58	X	Oliver Phillips; Tim Baker; Roel Brienen; Rodolfo Vasquez; Abel Monteagudo
SUC-04	-3.25	-72.89	1	698	264	135	X	Oliver Phillips; Tim Baker; Roel Brienen; Rodolfo Vasquez; Abel Monteagudo
SUC-05	-3.26	-72.89	1	660	204	110	X	Oliver Phillips; Tim Baker; Roel Brienen; Rodolfo Vasquez; Abel Monteagudo
SUC-10	-3.25	-72.91	0.08	49	40	35	-	Alwyn Gentry; Rodolfo Vasquez
TAM-01	-12.84	-69.29	1	953	209	122	X	Oliver Phillips; Tim Baker; Roel Brienen; Rodolfo Vasquez; Abel Monteagudo
TAM-02	-12.83	-69.29	1	1039	235	128	X	Oliver Phillips; Tim Baker; Roel Brienen; Rodolfo Vasquez; Abel Monteagudo
TAM-03	-12.84	-69.28	0.58	395	29	26	X	Oliver Phillips; Tim Baker; Roel Brienen; Rodolfo Vasquez; Abel Monteagudo

Plot Code	Lat.	Long.	Plot size ha	Nº Ind.	Nº Species	Nº Genera	Multiple census	Data contributors
TAM-04	-12.84	-69.28	0.42	458	182	106	X	Oliver Phillips; Tim Baker; Roel Brienen; Rodolfo Vasquez; Abel Monteagudo
TAM-05	-12.83	-69.27	1	891	210	115	X	Oliver Phillips; Tim Baker; Roel Brienen; Rodolfo Vasquez; Abel Monteagudo
TAM-06	-12.84	-69.3	1	904	226	128	X	Oliver Phillips; Tim Baker; Roel Brienen; Rodolfo Vasquez; Abel Monteagudo
TAM-07	-12.83	-69.26	1	923	194	112	X	Oliver Phillips; Tim Baker; Roel Brienen; Rodolfo Vasquez; Abel Monteagudo
TAM-08	-12.83	-69.27	1	663	158	96	X	Oliver Phillips; Tim Baker; Roel Brienen; Rodolfo Vasquez; Abel Monteagudo
TAM-09	-12.83	-69.28	1	594	174	118	X	Oliver Phillips; Yadvinder Malhi
TAM-20	-12.78	-69.28	0.1	57	32	29	-	Alwyn Gentry; Kenneth Young
TAM-21	-12.84	-69.29	0.1	75	38	32	-	Alwyn Gentry
TAM-22	-12.78	-69.28	0.1	65	38	32	-	Alwyn Gentry
TAM-23	-12.83	-69.27	0.1	80	47	38	-	Alwyn Gentry
TAN-02	-13.09	-52.38	1	549	53	41	X	Beatriz Marimon; Ben Hur Marimon Junior; Jon Lloyd; Oliver Phillips; Ted Feldpausch
TAN-03	-12.82	-52.36	1	641	53	38	X	Beatriz Marimon; Ben Hur Marimon Junior; Jon Lloyd; Oliver Phillips; Ted Feldpausch
TAN-04	-12.92	-52.37	1	660	60	44	X	Beatriz Marimon; Ben Hur Marimon Junior; Jon Lloyd; Oliver Phillips; Ted Feldpausch
TAP-50	-3.31	-54.94	0.25	180	63	54	X	Natalino Silva
TAP-51	-3.31	-54.94	0.25	159	60	51	X	Natalino Silva
TAP-52	-3.31	-54.94	0.25	159	68	59	X	Natalino Silva
TAP-53	-3.31	-54.94	0.25	130	54	47	X	Natalino Silva
TAP-54	-3.31	-54.95	0.25	153	57	45	X	Natalino Silva
TAP-55	-3.31	-54.95	0.25	152	58	49	X	Natalino Silva
TAP-56	-3.31	-54.95	0.25	146	58	52	X	Natalino Silva
TAP-57	-3.31	-54.95	0.25	128	59	49	X	Natalino Silva
TAP-58	-3.31	-54.94	0.25	154	57	51	X	Natalino Silva
TAP-59	-3.31	-54.94	0.25	153	66	55	X	Natalino Silva
TAP-60	-3.31	-54.94	0.25	133	58	49	X	Natalino Silva
TAP-61	-3.31	-54.94	0.25	141	62	54	X	Natalino Silva
TAY-01	11.33	-74.03	0.1	73	31	28	-	Alwyn Gentry
TEC-01	-1.71	-51.46	1	544	135	78	X	Leandro Ferreira
TEC-02	-1.74	-51.49	1	537	163	100	X	Leandro Ferreira
TEC-03	-1.73	-51.51	1	465	132	89	X	Leandro Ferreira
TEC-04	-1.75	-51.52	1	517	153	95	X	Leandro Ferreira
TEC-05	-1.78	-51.59	1	557	162	98	X	Leandro Ferreira
TEC-06	-1.73	-51.43	1	514	149	92	X	Leandro Ferreira
TEM-01	-2.97	-59.9	1	651	289	134	X	I• eda Amaral; Atila Alves

Plot Code	Lat.	Long.	Plot size ha	Nº Ind.	Nº Species	Nº Genera	Multiple census	Data contributors
TEM-02	-2.93	-59.95	1	643	261	129	X	I• eda Amaral; Atila Alves
TEM-03	-2.41	-59.9	1	705	267	126	X	I• eda Amaral; Atila Alves
TEM-04	-2.43	-59.79	1	655	260	125	X	I• eda Amaral; Atila Alves
TEM-05	-2.62	-60.21	1	725	292	139	X	I• eda Amaral; Atila Alves
TEM-06	-2.6	-60.11	1	751	274	124	X	I• eda Amaral; Atila Alves
TIP-01	-0.66	-76.4	1	702	166	106	X	Abel Monteagudo; Nigel Pitman; Roel Brienen
TIP-02	-0.63	-76.14	0.8	655	221	128	X	Abel Monteagudo David Neill, Oliver Phillips, Roel Brienen
TIP-03	-0.64	-76.15	1	605	134	93	X	Abel Monteagudo, David Neill, Oliver Phillips, Roel Brienen
TMP-01	-13.13	-69.57	2.25	1306	233	138	-	John Terborgh; Percy Núñez Vargas
TUT-01	5.76	-76.58	0.1	76	50	41	-	Alwyn Gentry
VCR-01	-14.83	-52.16	0.64	377	26	24	X	Beatriz Marimon; Ben Hur Marimon Junior; Ted Feldpausch; Jon Lloyd
VCR-02	-14.83	-52.17	0.6	338	59	51	X	Beatriz Marimon; Ben Hur Marimon Junior; Ted Feldpausch; Jon Lloyd
VCR-03	-14.83	-52.16	1	285	17	17	X	Beatriz Marimon; Ben Hur Marimon Junior; Ted Feldpausch; Jon Lloyd
VCR-04	-14.83	-52.17	1	485	73	57	X	Beatriz Marimon; Ben Hur Marimon Junior; Ted Feldpausch; Jon Lloyd
VEN-01	-4.67	-73.82	0.5	277	35	32	-	Eurídice Honorio; Fredy Rodriguez Dávila
VEN-02	-4.67	-73.82	0.5	346	28	27	-	Eurídice Honorio; Fredy Rodriguez Dávila
VTU-01	4.1	-66.63	0.1	79	33	29	-	Gerardo Aymard
VTU-02	4.1	-66.65	0.1	94	39	34	-	Gerardo Aymard
VTU-03	4.08	-66.62	0.1	131	19	19	-	Gerardo Aymard
VTU-04	4.18	-66.52	0.1	113	35	31	-	Gerardo Aymard
VTU-05	4.08	-66.72	0.1	119	37	33	-	Gerardo Aymard
VTU-06	4.13	-66.47	0.1	66	35	34	-	Gerardo Aymard
VTU-07	4.08	-66.43	0.1	53	21	21	-	Gerardo Aymard
VTU-08	4.7	-66.3	0.1	102	37	35	-	Gerardo Aymard
YAN-01	-3.43	-72.84	1	992	367	188	X	Oliver Phillips; Tim Baker; Roel Brienen; Rodolfo Vasquez; Abel Monteagudo
YAN-02	-3.43	-72.84	1	670	279	139	X	Oliver Phillips; Tim Baker; Roel Brienen; Rodolfo Vasquez; Abel Monteagudo
YAN-11	-3.43	-72.85	0.1	52	41	32	-	Alwyn Gentry; Rodolfo Vasquez
YAN-12	-3.43	-72.85	0.1	57	47	44	-	Alwyn Gentry; Rodolfo Vasquez
YAN-13	-3.46	-72.83	0.1	74	50	45	-	Alwyn Gentry; Rodolfo Vasquez
YUT-01	-2.35	-76.43	1	608	171	110	-	Kenneth Young; Ophelia Wang
ZAR-01	-4.01	-69.91	1	888	55	45	X	Eliana Jimenez, Jon Lloyd, Maria Peñuela, Oliver Phillips
ZAR-02	-4	-69.9	1	664	166	111	X	Eliana Jimenez, Jon Lloyd, Maria Peñuela, Oliver Phillips
ZAR-03	-3.99	-69.9	1	714	204	120	X	Eliana Jimenez, Jon Lloyd, Maria Peñuela, Oliver Phillips

Plot Code	Lat.	Long.	Plot size ha	Nº Ind.	Nº Species	Nº Genera	Multiple census	Data contributors
ZAR-04	-3.99	-69.91	1	667	129	84	X	Eliana Jimenez, Jon Lloyd, Maria Peñuela, Oliver Phillips
