# Supplementary material for: Evolutionary heritage influences Amazon tree ecology
Source: Proc Biol Sci. 2016 Dec 14;283(1844):20161587. doi: 10.1098/rspb.2016.1587 (PMC5204144; doi:10.1098/rspb.2016.1587)
Supplement: Methods for calculating trait intrinsic value [file rspb20161587supp2.pdf]

Proceedings of the Royal Society B

SUPPORTING INFORMATION

Evolutionary heritage influences Amazon tree ecology

Fernanda Coelho de Souza, Kyle G. Dexter, Oliver L. Phillips, Roel J.W. Brienen, Jerome Chave, David R. Galbraith, Gabriela Lopez-Gonzalez, Abel Monteagudo-Mendoza, R. Toby Pennington, Lourens Poorter, Miguel Alexiades, Esteban Álvarez-Dávila, Ana Andrade, Luis E.O.C. Aragão, Alejandro Araujo-Murakami, Eric J.M.M. Arets, Gerardo A. Aymard C., Christopher Baraloto, Jorcely Barroso, Damien Bonal, Rene G.A. Boot, José L.C. Camargo, James A. Comiskey, Fernando Cornejo Valverde, Plínio B. de Camargo, Anthony Di Fiore, Fernando Elias, Terry L. Erwin, Ted R. Feldpausch, Leandro Ferreira, Nykolas M.F. Fyllas, Emanuel Gloor, Bruno Herault, Rafael Herrera, Niro Higuchi, Eurídice N. Honorio Coronado, Timothy J. Killeen, William F. Laurance, Susan Laurance, Jon Lloyd, Thomas E. Lovejoy, Yadvinder Malhi, Leandro Maracahipes, Beatriz S. Marimon, Ben H. Marimon-Junior, Casimiro Mendoza, Paulo Morandi, David A. Neill, Percy Núñez Vargas, Edmar A. Oliveira, Eddie L. Oliveira, Walter A. Palacios, Maria C. Peñuela-Mora, John J. Pipoly III, Nigel C.A. Pitman, Adriana Prieto, Carlos A. Quesada, Hirma Ramirez-Angulo, Agustin Rudas, Kalle Ruokolainen, Rafael P. Salomão, Marcos Silveira, Juliana Stropp, Hans ter Steege, Raquel Thomas-Caesar, Peter van der Hout, Geertje M.F. van der Heijden, Peter J. van der Meer, Rodolfo V. Vasquez, Simone A. Vieira, Emilio Vilanova, Vincent A. Vos, Ophelia Wang, Kenneth R. Young, Roderick J. Zagt, Timothy R. Baker

Doi: 10.1098/rspb. 2016.1587

Additional Supporting information S2

We calculated ‘intrinsic’ trait values for potential tree size, mean and maximum growth rates and mortality rates using a mixed-effects modelling approach to account for the effect of variation in environmental conditions among plots (e.g. in precipitation, elevation and soil fertility) that affect plant traits [1-4]. We used models based on restricted maximum likelihood (REML), with plot (representing, for example, variation in topography, soil and/or climate) as a random effect and genus or species as the fixed effect in order to calculate genus-level ‘intrinsic’ values [5].

For potential tree size, the mean values for each genus-level fixed effect and their respective standard errors were used to calculate the 95th percentile of diameter, corrected for variation in environmental conditions. Mixed models were constructed separately for angiosperm trees and palms, as trees and palms have very distinctive growth patterns and physiological characteristics, which lead to different size-distribution patterns. Size distributions of palms are approximately normally distributed and therefore untransformed data was used for the mixed model analysis. However, the diameter distributions of trees are highly skewed to the right, and thus prior to the mixed model analysis, all diameter data was log-transformed to increase the normality of model residuals. Following analysis, estimates of maximum size for trees were then back transformed to be on the original scale.

Growth rates were similarly log-transformed prior to mixed model analysis. Due to the higher sampling needed to consistently estimate mortality rates [6], plots located in close proximity (with the same three letter code; table S1) were aggregated and

treated as having the same level in the random effect term in the mixed models of mortality rates, following Baker, Pennington [7].

Statistical analyses were performed in the R 3.1.1 [8] program using lme4 [9] and lmerBayes [10] packages.

1. Quesada C.A., Lloyd J., Schwarz M., Patiño S., Baker T.R., Czimczik C., Fyllas N.M., Martinelli L., Nardoto G.B., Schmerler J., et al. 2010 Variations in chemical and physical properties of Amazon forest soils in relation to their genesis. *Biogeosciences* **7**(5), 1515-1541. (doi:10.5194/bg-7-1515-2010).

2. Baker T.R., Phillips O.L., Malhi Y., Almeida S., Arroyo L., Di Fiore A., Erwin T., Higuchi N., Killeen T.J., Laurance S.G., et al. 2004 Increasing biomass in Amazonian forest plots. *Philos. Trans. R. Soc. Lond. Ser. B-Biol. Sci.* **359**(1443), 353-365. (doi:10.1098/rstb.2003.1422).

3. Quesada C.A., Phillips O.L., Schwarz M., Czimczik C.I., Baker T.R., Patiño S., Fyllas N.M., Hodnett M.G., Herrera R., Almeida S., et al. 2012 Basin-wide variations in Amazon forest structure and function are mediated by both soils and climate. *Biogeosciences* **9**(6), 2203-2246. (doi:10.5194/bg-9-2203-2012).

4. Baker T.R., Swaine M.D., Burslem D.F.R.P. 2003 Variation in tropical forest growth rates: combined effects of functional group composition and resource availability. *Perspect. Plant Ecol. Evol. Syst.* **6**(1-2), 21-36. (doi:10.1078/1433-8319-00040).

5. Fyllas N.M., Patino S., Baker T.R., Nardoto G.B., Martinelli L.A., Quesada C.A., Paiva R., Schwarz M., Horna V., Mercado L.M., et al. 2009 Basin-wide variations in foliar properties of Amazonian forest: phylogeny, soils and climate. *Biogeosciences* **6**(11), 2677-2708. (doi:10.5194/bg-6-2677-2009).

6. Rüger N., Huth A., Hubbell S.P., Condit R. 2011 Determinants of mortality across a tropical lowland rainforest community. *Oikos* **120**(7), 1047-1056. (doi:10.1111/j.1600-0706.2010.19021.x).

7. Baker T.R., Pennington R.T., Magallon S., Gloor E., Laurance W.F., Alexiades M., Alvarez E., Araujo A., Arets E.J., Aymard G., et al. 2014 Fast demographic traits promote high diversification rates of Amazonian trees. *Ecol. Lett.* **17**(5), 527-536. (doi:10.1111/ele.12252).

8. Team R.D.C. 2014 {R: A Language and Environment for Statistical Computing}.

9. Bates D., Mächler M., Bolker B., Walker S. 2015 Fitting Linear Mixed-Effects Models Usinglme4. *Journal of Statistical Software* **67**(1). (doi:10.18637/jss.v067.i01).

10. Condit R. 2012 CTFS R Package. (URL http://ctfs.arnarb.harvard.edu/Public/CTFSRPackage., Last accessed 15 April 2015.
