# Supplementary material for: Evolutionary heritage influences Amazon tree ecology
Source: Proc Biol Sci. 2016 Dec 14;283(1844):20161587. doi: 10.1098/rspb.2016.1587 (PMC5204144; doi:10.1098/rspb.2016.1587)
Supplement: Supporting reults [file rspb20161587supp3.pdf]

Proceedings of the Royal Society B

SUPPORTING INFORMATION

Evolutionary heritage influences Amazon tree ecology

Fernanda Coelho de Souza, Kyle G. Dexter, Oliver L. Phillips, Roel J.W. Brienen, Jerome Chave, David R. Galbraith, Gabriela Lopez-Gonzalez, Abel Monteagudo-Mendoza, R. Toby Pennington, Lourens Poorter, Miguel Alexiades, Esteban Álvarez-Dávila, Ana Andrade, Luis E.O.C. Aragão, Alejandro Araujo-Murakami, Eric J.M.M. Arets, Gerardo A. Aymard C., Christopher Baraloto, Jorcely Barroso, Damien Bonal, Rene G.A. Boot, José L.C. Camargo, James A. Comiskey, Fernando Cornejo Valverde, Plínio B. de Camargo, Anthony Di Fiore, Fernando Elias, Terry L. Erwin, Ted R. Feldpausch, Leandro Ferreira, Nykolas M.F. Fyllas, Emanuel Gloor, Bruno Herault, Rafael Herrera, Niro Higuchi, Eurídice N. Honorio Coronado, Timothy J. Killeen, William F. Laurance, Susan Laurance, Jon Lloyd, Thomas E. Lovejoy, Yadvinder Malhi, Leandro Maracahipes, Beatriz S. Marimon, Ben H. Marimon-Junior, Casimiro Mendoza, Paulo Morandi, David A. Neill, Percy Núñez Vargas, Edmar A. Oliveira, Eddie L. Oliveira, Walter A. Palacios, Maria C. Peñuela-Mora, John J. Pipoly III, Nigel C.A. Pitman, Adriana Prieto, Carlos A. Quesada, Hirma Ramirez-Angulo, Agustin Rudas, Kalle Ruokolainen, Rafael P. Salomão, Marcos Silveira, Juliana Stropp, Hans ter Steege, Raquel Thomas-Caesar, Peter van der Hout, Geertje M.F. van der Heijden, Peter J. van der Meer, Rodolfo V. Vasquez, Simone A. Vieira, Emilio Vilanova, Vincent A. Vos, Ophelia Wang, Kenneth R. Young, Roderick J. Zagt, Timothy R. Baker

Additional Supporting information S3

Comparison between different evolutionary models

Although continuous traits are typically assumed to evolve under Brownian motion (BM) [1], this may not be the most appropriate evolutionary model, and other models may perform better for estimating phylogenetic signal [2]. We compared the fit of the Brownian motion model of evolution with White-noise and lambda models. Brownian motion represents a random model of evolutionary change along each lineage, which assumes a constant rate of trait evolution through time, where covariance between genus trait values is proportional to the duration of their shared evolutionary history. Under pure BM, phylogenetic signal is equal to one. A white-noise model assumes that trait data come from a single normal distribution and there is no correlation between the ecological similarity and phylogenetic relatedness. A model including Pagel’s lambda represents a modification of a Brownian motion model and assesses the extent of phylogenetic signal by multiplying internal branches of the tree by the lambda parameter, which ranges from 0 to 1. When lambda values equal one, this model corresponds to BM.

Different evolutionary models were compared using the Akaikes’s information criterion AIC. For all traits, the model including Pagel’s lambda provided the best fit.

Table S3. Comparison of different evolutionary models considered for the evolution of: wood density, potential tree size, growth rates and mortality rates. The table shows lambda estimates and model fitting results statistic, with Akaike's Information Criterion (AIC) statistic for the different models: i) Lambda fits the extent to which phylogeny predict trait data; ii) Brownian Motion model of evolution; and iii) White-noise, stasis model with no phylogenetic signal, where phylogeny does not represents a good proxy for trait data

Trait		lambda λ	Lambda AIC	Brownian motion AIC	White-noise AIC
Wood density	wd	0.65	-9.07	198.75	173.73
Potential size	Max. D	0.49	424.48	582.30	497.29
	Max. Dwd	0.49	955.17	1112.34	1027.99
	Max. AGB	0.50	1129.23	1306.76	1215.75
Growth rates	Maxgr. D	0.40	488.48	632.05	520.77
	Maxgr. BA	0.42	770.89	888.98	809.07
	Maxgr. AGB	0.52	866.06	969.25	919.77
	Meangr. D	0.34	537.05	688.85	558.39
	Meangr. BA	0.36	754.16	894.83	785.57
	Meangr. AGB	0.44	733.03	858.56	782.41
Mortality	Mortality	0.39	478.60	596.46	491.45

References

1. Felsenstein J. 1985 Phylogenies and the Comparative Method. *The American Naturalist* **125**(1), 1-15. (doi:10.2307/2461605).

2. Münkemüller T., Lavergne S., Bzeznik B., Dray S., Jombart T., Schiffers K., Thuiller W. 2012 How to measure and test phylogenetic signal. *Methods in Ecology and Evolution* **3**(4), 743-756. (doi:10.1111/j.2041-210X.2012.00196.x).

Additional Supporting information S4

Table S4. Loadings of the first two axes of Phylogenetic Principal Components Analysis (PPCA) performed on wood density, potential tree size in diameter, maximum growth rate and mortality rate. All genera (n=214) which had a complete set of trait data were used for this analysis.

Trait	PPC1	PPC2
Maximum diameter	0.364	**0.909**
Maximum growth rates	**0.926**	0.195
Wood density	**-0.786**	0.123
Mortality	**0.711**	-0.584
Proportion of variance explained	52.80%	30.50%

* 83.3% of variance was explained by the first two axes.

Additional Supporting information S5

Table S5. Summary of trait data calculated across a subset of 26 plots (Manaus region), including number of individuals per genera, number of genera per trait and number of species. The table also shows the respective phylogenetic signal, for Manaus plots, Protieae, *Inga* and restricting the number of lineages to 214 genera that have values for all traits. Phylogenetic signal was measured using Blomberg's *K*. ***p<0.001; **p<0.05; *p<0.1.

Trait		Manaus					Burseraceae		Inga		Restricted number of lineages
		Nº ind	Nº Genera	Nº Species	Phylogenetic Signal (K)		Nº Species	Phylogenetic Signal (K)	Nº Species	Phylogenetic Signal (K)	
					Intrageneric variation						
					No	Yes					
Wood density	wd	-	292	293	0.25***	0.27***	23	0.55	27	0.21	0.24***
Potential size	Max. D	33.453	160	364	0.26***	0.40***	37	0.34	40	0.3	0.23**
	Max. Dwd	33.453	160	364	0.29***	0.44***	37	0.23	40	0.24	0.25***
	Max. BA	33.453	160	364	0.26***	0.40***	37	0.34	40	0.3	0.23***
	Max. AGB	33.453	160	364	0.29***	0.45***	37	0.24	40	0.31	0.24***
Growth rates	Max. gr. D	27.327	152	315	0.25**	0.41***	31	0.35	29	0.7	0.25**
	Max. gr. BA	27.327	152	315	0.26***	0.37***	31	0.33	29	0.86**	0.2**
	Max. gr. AGB	27.327	152	315	0.28***	0.39***	31	0.2	29	0.52	0.22**
	Mean. gr. D	27.327	152	315	0.28***	0.45***	30	0.33	29	0.69	0.26***
	Mean.gr. BA	27.327	152	315	0.28***	0.41***	30	0.38	29	0.76*	0.19*
	Mean.gr.AGB	27.327	152	315	0.30***	0.45***	30	0.16	29	0.4	0.24***
Mortality	Mortality	26.894	67	69	0.32***	0.43***	-	-	-	-	0.24***
