# Supplementary material for: Evolutionary heritage influences Amazon tree ecology
Source: Proc Biol Sci. 2016 Dec 14;283(1844):20161587. doi: 10.1098/rspb.2016.1587 (PMC5204144; doi:10.1098/rspb.2016.1587)

Proceedings of the Royal Society B

SUPPORTING INFORMATION

Evolutionary heritage influences Amazon tree ecology

Fernanda Coelho de Souza, Kyle G. Dexter, Oliver L. Phillips, Roel J.W. Brienen, Jerome Chave, David R. Galbraith, Gabriela Lopez-Gonzalez, Abel Monteagudo-Mendoza, R. Toby Pennington, Lourens Poorter, Miguel Alexiades, Esteban Álvarez-Dávila, Ana Andrade, Luis E.O.C. Aragão, Alejandro Araujo-Murakami, Eric J.M.M. Arets, Gerardo A. Aymard C., Christopher Baraloto, Jorcely Barroso, Damien Bonal, Rene G.A. Boot, José L.C. Camargo, James A. Comiskey, Fernando Cornejo Valverde, Plínio B. de Camargo, Anthony Di Fiore, Fernando Elias, Terry L. Erwin, Ted R. Feldpausch, Leandro Ferreira, Nykolas M.F. Fyllas, Emanuel Gloor, Bruno Herault, Rafael Herrera, Niro Higuchi, Eurídice N. Honorio Coronado, Timothy J. Killeen, William F. Laurance, Susan Laurance, Jon Lloyd, Thomas E. Lovejoy, Yadvinder Malhi, Leandro Maracahipes, Beatriz S. Marimon, Ben H. Marimon-Junior, Casimiro Mendoza, Paulo Morandi, David A. Neill, Percy Núñez Vargas, Edmar A. Oliveira, Eddie L. Oliveira, Walter A. Palacios, Maria C. Peñuela-Mora, John J. Pipoly III, Nigel C.A. Pitman, Adriana Prieto, Carlos A. Quesada, Hirma Ramirez-Angulo, Agustin Rudas, Kalle Ruokolainen, Rafael P. Salomão, Marcos Silveira, Juliana Stropp, Hans ter Steege, Raquel Thomas-Caesar, Peter van der Hout, Geertje M.F. van der Heijden, Peter J. van der Meer, Rodolfo V. Vasquez, Simone A. Vieira, Emilio Vilanova, Vincent A. Vos, Ophelia Wang, Kenneth R. Young, Roderick J. Zagt, Timothy R. Baker

Doi: 10.1098/rspb. 2016.1587

Additional Supporting information S6

Wood density

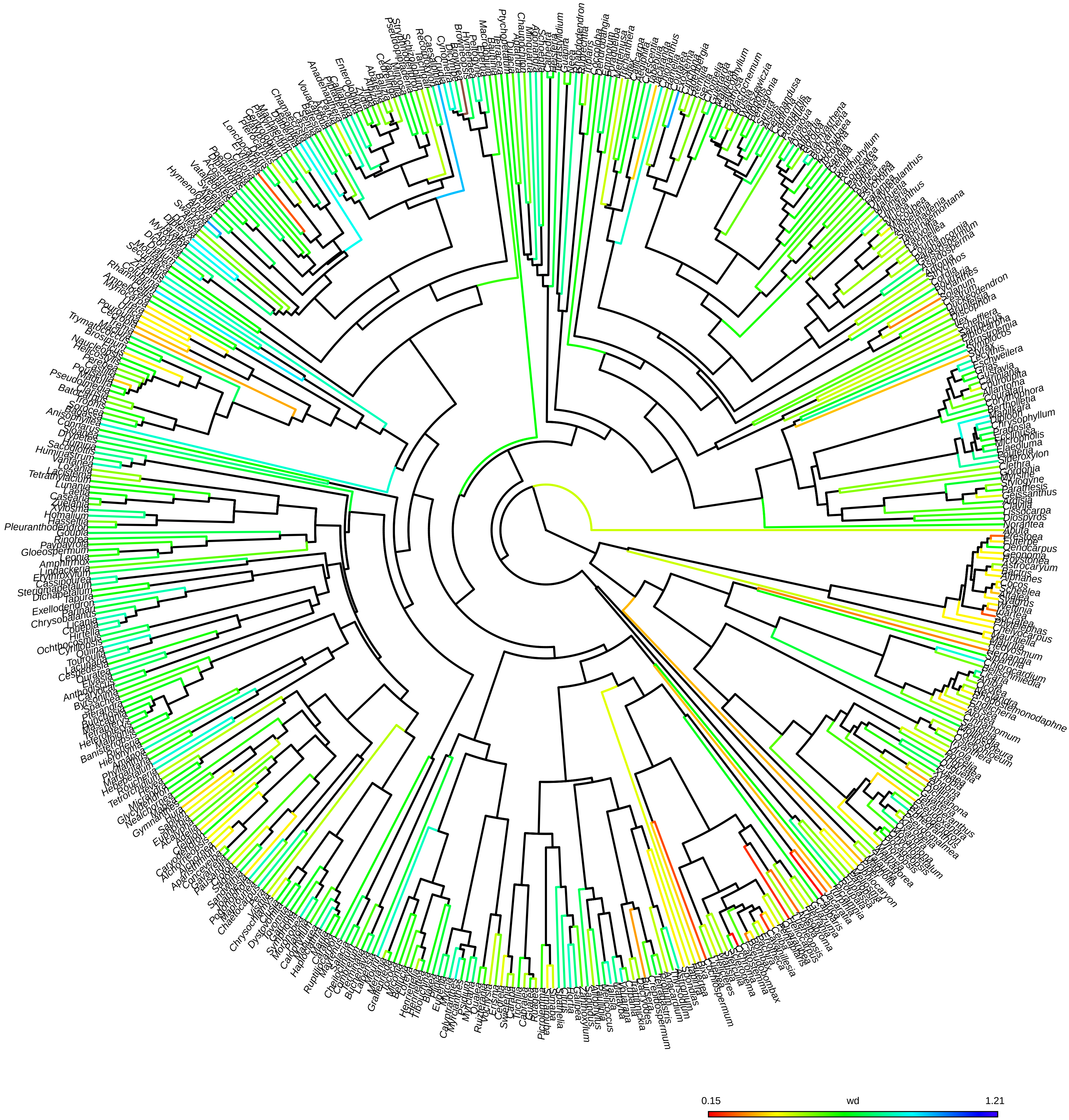

Maximum D

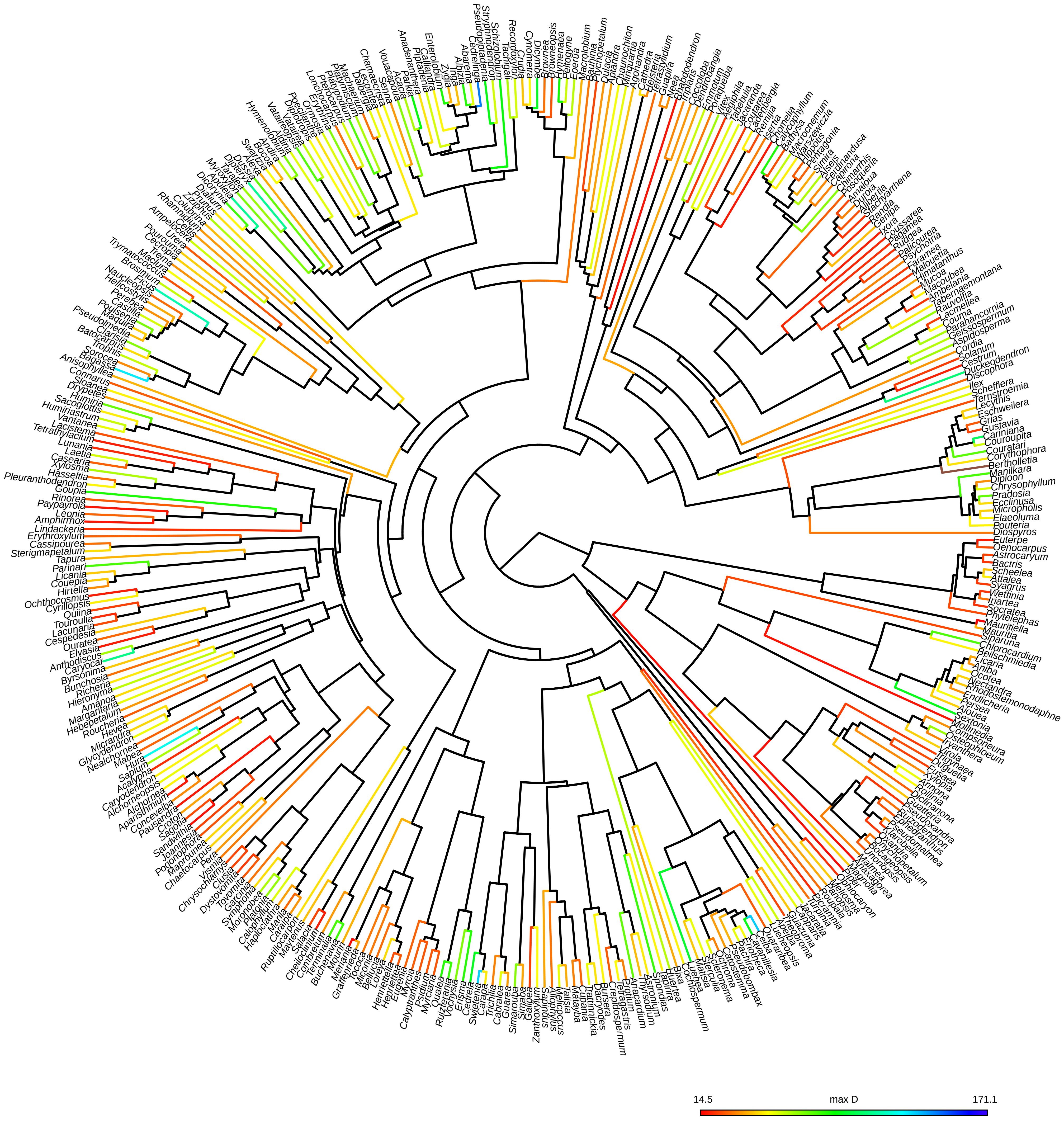

Maximum Growth D

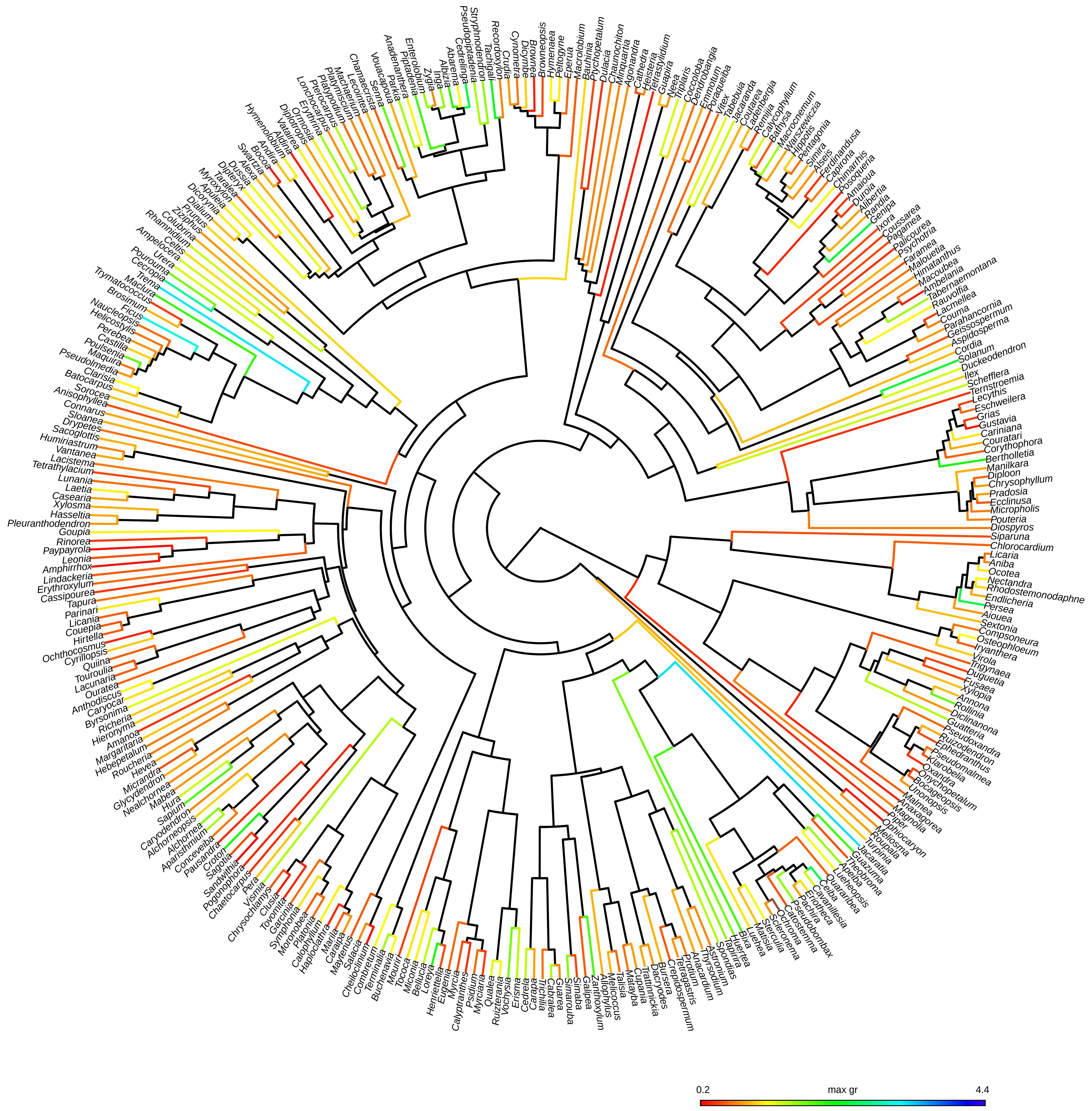

Mortality rate

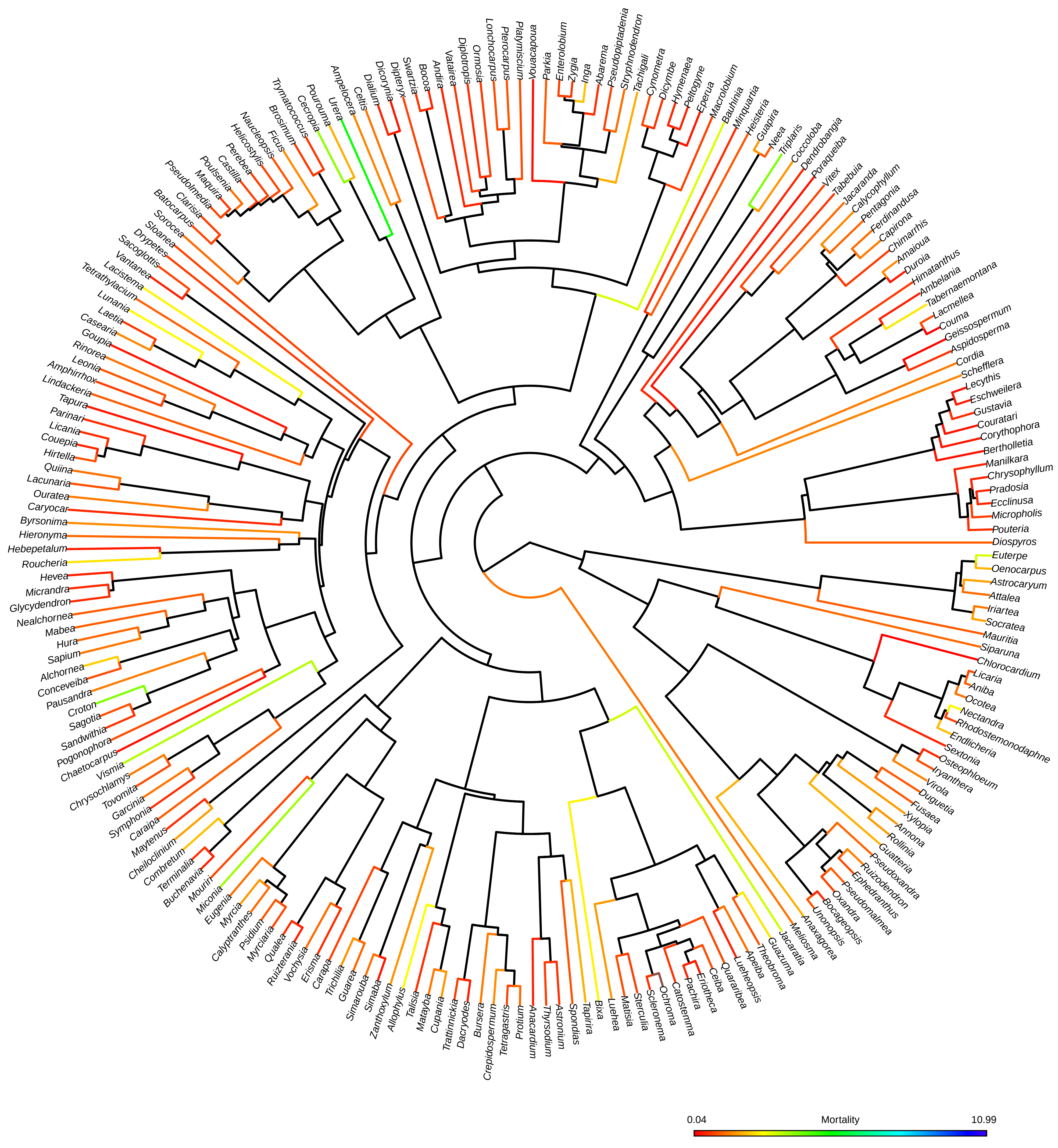

Supplement: Phylogeny of 497 Amazonian tree genera [file rspb20161587supp4.pdf]
