# Supplementary material for: Evolutionary heritage influences Amazon tree ecology
Source: Proc Biol Sci. 2016 Dec 14;283(1844):20161587. doi: 10.1098/rspb.2016.1587 (PMC5204144; doi:10.1098/rspb.2016.1587)
Supplement: Comparison between published values of phylogenetic signal and values found in the present study [file rspb20161587supp5.pdf]

Proceedings of the Royal Society B

SUPPORTING INFORMATION

Evolutionary heritage influences Amazon tree ecology

Fernanda Coelho de Souza, Kyle G. Dexter, Oliver L. Phillips, Roel J.W. Brienen, Jerome Chave, David R. Galbraith, Gabriela Lopez-Gonzalez, Abel Monteagudo-Mendoza, R. Toby Pennington, Lourens Poorter, Miguel Alexiades, Esteban Álvarez-Dávila, Ana Andrade, Luis E.O.C. Aragão, Alejandro Araujo-Murakami, Eric J.M.M. Arets, Gerardo A. Aymard C., Christopher Baraloto, Jorcely Barroso, Damien Bonal, Rene G.A. Boot, José L.C. Camargo, James A. Comiskey, Fernando Cornejo Valverde, Plínio B. de Camargo, Anthony Di Fiore, Fernando Elias, Terry L. Erwin, Ted R. Feldpausch, Leandro Ferreira, Nykolas M.F. Fyllas, Emanuel Gloor, Bruno Herault, Rafael Herrera, Niro Higuchi, Eurídice N. Honorio Coronado, Timothy J. Killeen, William F. Laurance, Susan Laurance, Jon Lloyd, Thomas E. Lovejoy, Yadvinder Malhi, Leandro Maracahipes, Beatriz S. Marimon, Ben H. Marimon-Junior, Casimiro Mendoza, Paulo Morandi, David A. Neill, Percy Núñez Vargas, Edmar A. Oliveira, Eddie L. Oliveira, Walter A. Palacios, Maria C. Peñuela-Mora, John J. Pipoly III, Nigel C.A. Pitman, Adriana Prieto, Carlos A. Quesada, Hirma Ramirez-Angulo, Agustin Rudas, Kalle Ruokolainen, Rafael P. Salomão, Marcos Silveira, Juliana Stropp, Hans ter Steege, Raquel Thomas-Caesar, Peter van der Hout, Geertje M.F. van der Heijden, Peter J. van der Meer, Rodolfo V. Vasquez, Simone A. Vieira, Emilio Vilanova, Vincent A. Vos, Ophelia Wang, Kenneth R. Young, Roderick J. Zagt, Timothy R. Baker

Doi: 10.1098/rspb. 2016.1587

Additional Supporting information S7

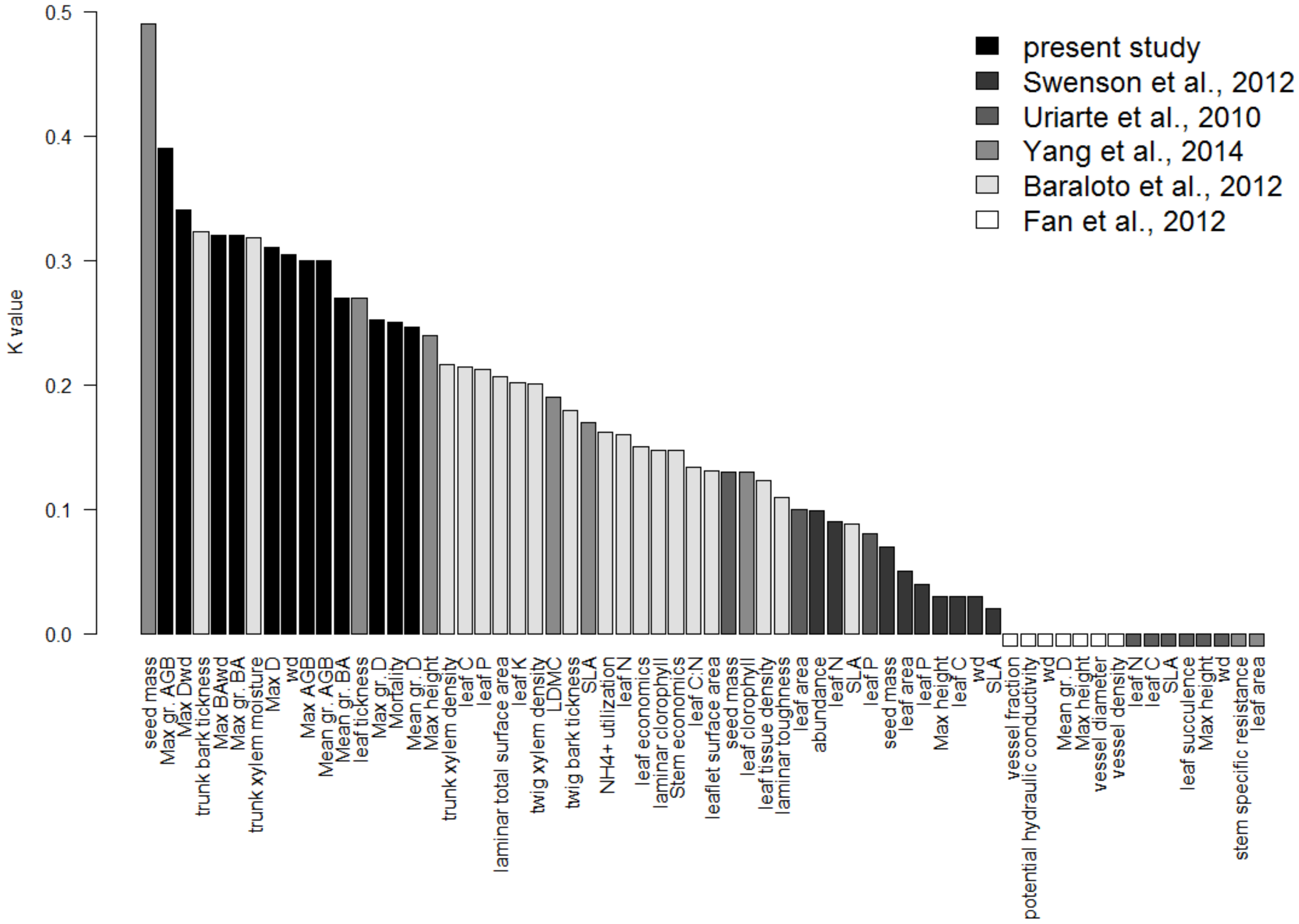

Figure S7. Comparison between published values of phylogenetic signal for traits of tropical forest trees measured by Blomberg's K statistic. Bar colour indicates the different studies [1-5]; bars in black are represented by traits calculated in the present study and shades of grey show values for published studies. Bars with negative values represent absence of phylogenetic signal.

1. Swenson N.G., Stegen J.C., Davies S.J., Erickson D.L., Forero-Montana J., Hurlbert A.H., Kress W.J., Thompson J., Uriarte M., Wright S.J., et al. 2012 Temporal turnover in the composition of tropical tree communities: functional determinism and phylogenetic stochasticity. *Ecology* **93**(3), 490-499.
2. Uriarte M., Swenson N.G., Chazdon R.L., Comita L.S., John Kress W., Erickson D., Forero-Montana J., Zimmerman J.K., Thompson J. 2010 Trait similarity, shared ancestry and the structure of neighbourhood interactions in a subtropical wet forest: implications for community assembly. *Ecol. Lett.* **13**(12), 1503-1514. (doi:10.1111/j.1461-0248.2010.01541.x).
3. Yang J., Zhang G., Ci X., Swenson N.G., Cao M., Sha L., Li J., Baskin C.C., Slik J.W.F., Lin L., et al. 2014 Functional and phylogenetic assembly in a Chinese tropical tree community across size classes, spatial scales and habitats. *Funct. Ecol.* **28**(2), 520-529. (doi:10.1111/1365-2435.12176).
4. Baraloto C., Hardy O.J., Paine C.E.T., Dexter K.G., Cruaud C., Dunning L.T., Gonzalez M.-A., Molino J.-F., Sabatier D., Savolainen V., et al. 2012 Using functional traits and phylogenetic trees to examine the assembly of tropical tree communities. *J. Ecol.* **100**(3), 690-701. (doi:10.1111/j.1365-2745.2012.01966.x).
5. Fan Z.-X., Zhang S.-B., Hao G.-Y., Ferry Slik J.W., Cao K.-F. 2012 Hydraulic conductivity traits predict growth rates and adult stature of 40 Asian tropical tree species better than wood density. *J. Ecol.* **100**(3), 732-741. (doi:10.1111/j.1365-2745.2011.01939.x).
